# Radical Fluoromethylation Enabled by Cobalamin-Dependent Radical SAM Enzymes

**DOI:** 10.1021/acsbiomedchemau.5c00062

**Published:** 2025-05-06

**Authors:** Syam Sundar Neti, Bo Wang, Jiayuan Cui, David F. Iwig, Nicholas J. York, Anthony J. Blaszczyk, Matthew R. Bauerle, Squire J. Booker

**Affiliations:** † Department of Chemistry, 8082 The Pennsylvania State University, University Park, Pennsylvania 16802, United States; ‡ Department of Biochemistry and Molecular Biology, The Pennsylvania State University, University Park, Pennsylvania 16802, United States; § Howard Hughes Medical Institute, The Pennsylvania State University, University Park, Pennsylvania 16802, United States

**Keywords:** *S*-adenosyl-l-homocysteine (SAH), halide methyltransferase (HMT), fluoromethyl iodide (FMeI), iron−sulfur clusters, cobalamin, radical SAM (RS), *S*-adenosylmethionine (SAM)

## Abstract

Fluorine is an important atom in many drugs because it can improve the efficacy and metabolic stability of many molecules. Strategies to incorporate monofluoromethyl groups in drugs have been limited and have received less attention than strategies for difluoromethylation or trifluoromethylation. Previously, we and others reported the enzymatic monofluoromethylation of several biologically relevant metabolites based on the transfer of a fluoromethyl group from analogs of *S*-adenosylmethionine (SAM) to various nucleophiles (carbon, oxygen, nitrogen, sulfur, and carbon) through a polar S_N_2 mechanism. However, this strategy is limited to molecules containing nucleophilic target atoms. Inspired by a subset of enzymes within the radical SAM superfamily that can methylate inert carbon atoms, we developed an enzymatic strategy to transfer fluoromethyl groups to unactivated carbon atoms. This strategy leverages the ability of halide methyltransferase to generate a transient fluoromethyl-containing SAM analog. Our studies show that *S*-adenosyl-*L*-(fluoromethyl)­methionine can undergo reductive cleavage to a 5′-deoxyadenosyl 5′-radical, which initiates radical-dependent fluoromethylation through substrate hydrogen-atom abstraction. Adding fluoromethyl groups to unactivated C–H bonds using radical SAM enzymes is a powerful approach that can be used to derivatize molecules of interest where S_N_2-based fluoromethylation is precluded.

## Graphical abstract

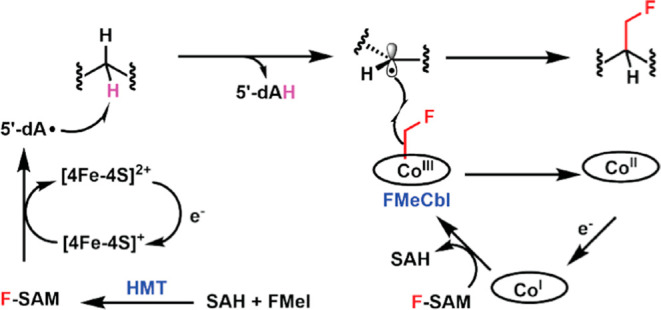

## Introduction

Fluorine atoms are found in 20%–30% of all pharmaceuticals and ∼30% of all agrochemicals.^1–6^ Because of its electronegativity and relatively small size, fluorine can substantially alter the physicochemical properties of an entire molecule, including lipophilicity, metabolic stability, cell permeability, and pharmacokinetic profiles.^2,4,6–8^ In addition, fluorine-containing compounds can be studied by ^19^F nuclear magnetic resonance (NMR) spectroscopy with low noise and high sensitivity to probe the molecular properties of drug-target interactions.^9^ ^18^F-labeled compounds are also widely used in disease diagnosis by positron emission tomography.^10^ Because of fluorine’s importance in pharmacology and agroscience, chemists have developed myriad strategies to incorporate fluorine into molecules, including direct fluorination of C–H bonds,^11^ fluorine substitution of functional groups,^12^ and transferring fluorine-containing fragments (mono-, di-, and trifluoromethyl groups).^13^

Monofluoromethyl groups (FMe) are particularly interesting because they occur in many drug molecules (Figure 1A). FMe is bioisosteric to several important functional groups, including methyl, hydroxymethyl, and aminomethyl.^7,14^ The importance of FMe groups in drug development is well recognized and has been a focus of synthetic chemists for many years.^15–21^ However, synthetic methods for incorporating FMe groups have several disadvantages, such as toxic monofluoromethylating reagents and the lack of robust regio-, chemo-, or stereoselectivity. Biocatalysts have the potential to address these disadvantages due to their excellent selectivities and mild reaction conditions.^22,23^

**Figure 1:**
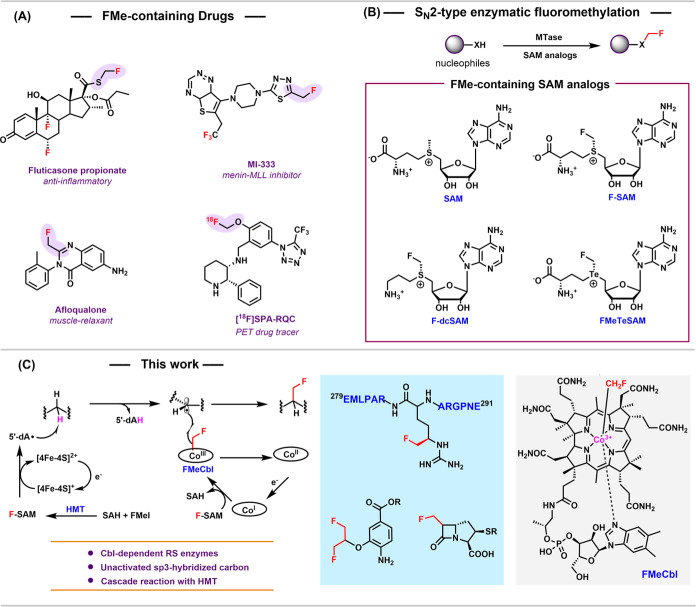
(A) Select FMe-containing drugs; (B) MTase-catalyzed polar fluoromethylation and chemical structures of SAM and SAM analogs for fluoromethylation; (C) Left panel: B_12_-RSMTs catalyzing radical fluoromethylation; Middle panel: fluoromethylated products in this work; Right panel: chemical structure of FMeCbl.

Methyltransferases (MTases) were recently explored as biocatalysts for enzymatic monofluoromethylation.^24–26^ Most MTases use *S*-adenosylmethionine (SAM) to append methyl groups to various nucleophilic acceptors (e.g., –O, –S, –N, –C, -Se, Te, and As) in many biological molecules.^27^ Canonical MTase-enabled methylation proceeds via an S_N_2 mechanism where the activated methyl group of SAM serves as an electrophilic methyl donor. Compared with synthetic methods, MTase-catalyzed methylation is highly regioselective, making it a promising biocatalytic strategy for the late-stage diversification of complex molecules.^28,29^

Seebeck et al. reported the *in situ* generation of *S*-adenosyl-*L*-(fluoromethyl)­homocysteine (F-SAM) and its application in transferring FMe groups to various nucleophiles (Figure 1B).^26^ F-SAM, generated using fluoromethyl iodide, *S*-adenosylhomocysteine (SAH), and halide methyltransferase (HMT), is not sufficiently stable to isolate. Its use requires it to be generated in a cascade reaction with an appropriate MTase to effect FMe transfer. F-SAM decomposes through an intramolecular attack of its carboxylate group on the C_γ_ of its methionyl.^24,30^ To address this stability issue, Dong et al. synthesized a decarboxylated version of F-SAM (F-dcSAM).^24^ F-dcSAM exhibited a higher stability than F-SAM, and several MTases could use it to transfer FMe groups. Booker et al. designed and synthesized a tellurium-containing analog of SAM, *Te*-adenosyl-*L*-(fluoromethyl)­homotellurocysteine (FMeTeSAM).^25^ FMeTeSAM showed excellent stability and was also accepted by several MTases to transfer FMe groups to oxygen, nitrogen, sulfur, and some carbon nucleophiles. These three studies provided a significant advance in introducing fluorine into complex molecules biocatalytically. However, they were limited to S_N_2 reactions, leaving the fluoromethylation of unactivated carbon atoms an unexplored chemical space.

Radical *S*-adenosylmethionine (RS) methylases (RSMTs) are an emerging class of enzymes that append methyl groups on unactivated carbon atoms.^31–37^ Those that employ a cobalamin (B_12_ or Cbl) cofactor in addition to the universal [Fe_4_S_4_] cluster constitute the largest subclass of these enzymes.^36,38,39^ They use the oxidizing power of a 5′-dA^•^, generated from the reductive cleavage of SAM, to abstract hydrogen atoms (H^•^) from the substrate to yield a substrate-based radical. A methyl group is transferred from SAM to cob­(I)­alamin via an S_N_2 process to form methylcobalamin (MeCbl). Next, the substrate radical attacks MeCbl, inducing a homolytic cleavage of the Co–Me bond and resulting in the transfer of a methyl group to the substrate. A handful of Cbl-dependent RS enzymes have been characterized, primarily involved in natural product biosynthesis.^40–47^ However, using Cbl-dependent RS enzymes as biocatalysts has been underdeveloped, especially in FMe transfer. Herein, we report radical-dependent fluoromethylation enabled by several Cbl-dependent RS methylases (B_12_-RSMTs) coupled with HMT to generate F-SAM *in situ* (Figure 1C). We note that while this manuscript was being finalized, Kong et al. also reported a similar strategy to effect radical-dependent fluoromethylation.^48^

## Materials and Methods

### Materials

*N*-(2-Hydroxyethyl)-piperazine-*N*′-(2-ethanesulfonic acid) (HEPES) was purchased from Fisher Scientific. Imidazole was purchased from J. T. Baker Chemical Co., and potassium chloride and glycerol from EMD Chemicals. β-Mercaptoethanol (BME), 5′-deoxyadenosine (5′-dAH) and *S*-adenosylhomocysteine (SAH) were purchased from MilliporeSigma. FMeI and MeI were purchased from Acros-Organics. Kanamycin, ampicillin, dithiothreitol (DTT), arabinose, isopropyl β-D-1-thiogalactopyranoside (IPTG), and tris­(2-carboxyethyl)­phosphine hydrochloride (TCEP) were purchased from Gold Biotechnology. Ni-nitrilotriacetic acid (NTA) resin was acquired from Qiagen. SAM was synthesized and purified as described previously.^30^ DNA isolation kits were purchased from Macherey-Nagel (Dueren, Germany). All other chemicals and materials were of the highest grade available and were from MilliporeSigma.

CysS substrates and products (OMe, OEt, O^*i*^Pr, O^*s*^Bu, O^*t*^Bu) were chemically synthesized as described in the Supporting Information. The Mmp10 13-mer peptide (^279^EMLPAR**R**ARGPNE^291^) substrate surrogate was purchased from Think Peptides (www.thinkpeptides.com). The substrate for the TokK reaction, used in a previous publication, was provided by Dr. Craig Townsend (Johns Hopkins University).^43^

## General Methods

UV–visible spectra were recorded on a Varian Cary 50 spectrometer (Agilent, Walnut Creek, CA) using the WinUV software package to control the instrument. High-performance liquid chromatography (HPLC) with detection by tandem mass spectrometry (LC–MS) was conducted on a Thermo Scientific Vanquish UHPLC system coupled to a Thermo Scientific Q Exactive HF-X mass spectrometer.

### Overproduction and Purification of Mmp10 and CysS and Variants

The *Mammp10* gene was cloned as previously reported.^49^ The gene encoding CysS (Corallococcus sp. *CA054B* (UniProt ID: A0A3A8HCN5, *cc*CysS)) was codon-optimized for expression in Escherichia coli and cloned into a pET28a­(+) vector using *Nde*I/*Xho*I restriction sites. The resulting pET-28a­(+)-Mmp10 plasmid was used to transform E. coli BL21 (DE3) competent cells containing the pDB1282 plasmid, which harbors the *isc* operon from Azotobacter vinelandii.^50^ Overproduction and purification of Mmp10 was performed as described previously.^49,51^

The pET-28a­(+)-CysS plasmid was used to transform E. coli BL21 (DE3) competent cells containing the pDB1282 and pBAD42-BtuCEDFB plasmids. The pBAD42-BtuCEDFB plasmid contains genes for cobalamin uptake and transport into the cell.^50,52^ Site-directed mutagenesis was performed using the pET-28a­(+)-CysS plasmid as a template with the primers shown in Table S1. The resulting sequence changes were verified by DNA sequencing at the Penn State Genomics Core Facility (University Park, PA), and the resulting plasmids were used to transform E. coli BL21 (DE3) competent cells. Primers used for site-directed mutagenesis were purchased from Integrated DNA Technologies (Coralville, IA).

A 200 mL starter culture in lysogeny broth (LB) containing 50 mg/L kanamycin, 100 mg/L ampicillin, and 50 mg/L spectinomycin was inoculated with a single colony of either WT CysS or the corresponding variant and incubated at 37 °C with shaking at 250 rpm for 12 h. Twenty mL of the starter culture was used to inoculate 4 L of ethanolamine M9 medium containing 50 mg/L kanamycin, 100 mg/L ampicillin, and 50 mg/L spectinomycin and incubated at 37 °C with shaking at 180 rpm.^53^ Expression of the genes encoded on plasmid pDB1282 was induced at an OD_600_ of 0.3 with arabinose (8.0 g, 0.2% w/v). At an OD_600_ of 0.6, the flasks were placed in an ice-water bath for 30 min. Once cooled, IPTG was added to a final concentration of 0.5 mM, and iron­(III) chloride (FeCl_3_) was added to a final concentration of 25 μM. The cultures were incubated for 18 h at 18 °C with shaking at 150 rpm. The cells were harvested, flash-frozen in liquid nitrogen, and stored at −80 °C until use.

45 g of frozen cell paste was brought into an anaerobic chamber containing <1 ppm of O_2_ (Coy Laboratory products, Grass Lake, Michigan). The cells were resuspended in 150 mL of lysis buffer (50 mM HEPES, pH 7.5, 300 mM KCl, 10% (v/v) glycerol, 2.5 mM imidazole, and 10 mM BME) containing lysozyme (1 mg/mL), DNaseI (0.1 mg/mL), and EDTA-free protease inhibitor tablet. The protein was reconstituted with the [Fe_4_S_4_] cluster and hydroxycobalamin (OHCbl) cofactors during the cell lysis by supplementing with hydroxocobalamin acetate (1 mg/g of cell paste), cysteine (1 mg/mL), FeCl_3_ (0.8 mg/mL), and pyridoxal 5′-phosphate (1 mg/g of cell paste). The suspension was stirred at room temperature for 30 min to generate a homogeneous mixture. The resuspended cells were incubated on ice and subjected to six 45 s sonic bursts (50% output) on a QSonica instrument in a Coy anaerobic chamber with 8 min intermittent pauses. The lysate was centrifuged for 1 h at 50,000*g* at 4 °C. The resulting supernatant was loaded onto Ni-NTA resin equilibrated in the lysis buffer. The resin was washed with 200 mL of the wash buffer (50 mM HEPES, pH 7.5, 300 mM KCl, 10% (v/v) glycerol, 20 mM imidazole, 10 mM BME) followed by elution of the protein with elution buffer (50 mM HEPES, pH 7.5, 300 mM KCl, 250 mM imidazole, 10% (v/v) glycerol, and 10 mM BME). The eluted protein was concentrated by ultracentrifugation using an Amicon Ultra centrifugal filter unit with a 10 kDa molecular weight cutoff membrane (MilliPore). The protein was exchanged into S200 buffer (50 mM HEPES, pH 7.5, 300 mM KCl, 10 mM imidazole, 10% (v/v) glycerol, and 1.5 mM BME) using a PD-10 column (GE Healthcare). Chemical reconstitution of CysS or its variants was performed overnight on ice in 200 mM HEPES buffer, pH 7.5, containing 0.1 mM CysS or variant, 0.2 mM Cbl, 0.4 mM FeCl_3_, 0.4 mM Na_2_S, and 5 mM DTT. The resulting reconstituted protein was further purified by size-exclusion chromatography on a HiPrep 26/60 S200 column (Cytiva) housed in an anaerobic chamber, eluting isocratically in S200 buffer (50 mM HEPES, pH 7.5, 400 mM KCl, 10% glycerol, and 4 mM DTT). Fractions containing monomeric CysS were pooled, concentrated, frozen, and stored in liquid nitrogen. Protein homogeneity was judged by sodium dodecyl sulfate–polyacrylamide gel electrophoresis (SDS-PAGE), and protein concentration was determined by the method of Bradford, using bovine serum albumin (fraction V) as a standard.

### Quantification of Cbl and Iron in CysS and Its Variants

The concentrations of protein-bound Cbl were determined by UV–vis spectroscopy by treating the protein with potassium cyanide to form a dicyano-cobalamin adduct as previously described.^54^ Colorimetric iron analyses were conducted on the purified protein using the methods of Beinert.^55–57^

### Overproduction and Purification of TokK, Fom3, and Halide Methyl Transferase (HMT)

Overproduction and purification of TokK,^43^ Fom3,^58^ and halide methyl transferase (HMT)^26^ were performed as described previously.

### LC–MS Activity Assays of Mmp10

Activity measurements were conducted in a Coy anaerobic chamber containing <1 ppm of O_2_. Each reaction contained the following at their final concentrations in a final volume of 700 μL: 100 mM HEPES, pH 7.5, 100 mM KCl, 15 μM Mmp10, 0.4 mM SAH, 10 mM FMeI/MeI, 100 M HMT, 0.5 mM 13-mer peptide substrate, 1.5 mM of titanium­(III) citrate (TiCi), and 100 μM l-tryptophan (internal standard). Reactions were initiated by adding 10 mM (final concentration) FMeI/MeI, and 25 μL aliquots were removed at various times and added to an equal volume of 200 mM H_2_SO_4_ to quench the reaction. Reactions were run in triplicate. The samples were centrifuged at 14,000*g* for 30 min, and their supernatants were used for further analysis. The time-dependent formation of methylated and fluoromethylated peptide product, 5′-dAH, FMe- and MeCbl, as well as the decay of the 13-*mer*-peptide substrate and SAH were determined by high-resolution LC–MS. Detection of substrates and products was performed using electrospray ionization (ESI^+^) in positive mode, and quantification was based on standard curves of substrates and products. The assay mixture was separated on an Agilent Technologies Zorbax Extend-C18 column Rapid Resolution HT (4.6 mm × 50 mm, 1.8 μm particle size) equilibrated in 98% Solvent A (0.1% formic acid, pH 2.6) and 2% Solvent B (0.1% formic acid in 100% acetonitrile). A gradient of 2–100% Solvent B was applied from 2 to 10 min and maintained at 100% Solvent B until 12 min before returning to 2% Solvent B from 12 to 13.5 min. A flow rate of 0.3 mL/min was maintained throughout the method. The column was allowed to re-equilibrate for an additional 2 min under the initial conditions between sample injections.

### LC–MS Activity Assays of CysS and Variants

Activity measurements were conducted in a Coy anaerobic chamber containing <1 ppm of O_2_. Each reaction contained the following at their final concentrations in a final volume of 700 μL: 75 mM HEPES, pH 7.5, 250 mM KCl, 50 μM CysS, 0.4 mM SAH, 10 mM FMeI/MeI, 100 M HMT, 0.5 mM of -OMe or -OEt or -O^i^Pr substrate, 2 mM of TiCi, and 90 μM l-tryptophan (internal standard). Reactions were initiated by adding 10 mM (final concentration) FMeI or MeI, and 30 μL aliquots were removed at various times and added to an equal volume of MeOH to quench the reaction. Reactions were run in triplicate. The samples were centrifuged at 14,000*g* for 30 min, and the supernatants were used for further analysis. The time-dependent formation of methylated and fluoromethylated products, 5′-dAH, FMe- and MeCbl, as well as the decay of the -OMe or -OEt or -O^i^Pr substrate and SAH were determined by high-resolution LC–MS. For the CysS variants, only the -OMe substrate was assayed for the detection of methylated and fluoromethylated products to compare the efficiency of fluoromethylation relative to WT CysS. The detection of substrates and products was performed using electrospray ionization (ESI^+^) in positive mode, and quantification was based on standard curves of substrates and products. The assay mixture was separated on an Agilent Technologies Zorbax Extend-C18 column Rapid Resolution HT (4.6 mm × 50 mm, 1.8 μm particle size) equilibrated in 98% Solvent A (0.1% formic acid, pH 2.6) and 2% Solvent B (0.1% formic acid in 100% acetonitrile). A gradient of 2–100% Solvent B was applied from 2 to 14 min and maintained at 100% Solvent B until 17 min before returning to 2% Solvent B from 17 to 18.5 min. A flow rate of 0.3 mL/min was maintained throughout the method. The column was allowed to re-equilibrate for an additional 2.5 min under the initial conditions between sample injections.

### LC–MS Activity Assays of TokK

Activity measurements were conducted in a Coy anaerobic chamber containing <1 ppm of O_2_. Each reaction contained the following at their final concentrations in a final volume of 700 μL: 100 mM HEPES, pH 7.5, 200 mM KCl, 55 μM Tokk, 0.4 mM SAH, 10 mM FMeI/MeI, 100 M HMT, 0.15 mM carbapenam substrate, 0.2 mM OHCbl, 1.5 mM of TiCi, and 90 μM l-tryptophan (internal standard). Reactions were initiated by adding 10 mM (final concentration) FMeI or MeI, and 30 μL aliquots were removed at various times and added to an equal volume of MeOH to quench the reaction. Reactions were run in triplicate. The samples were centrifuged at 14,000*g* for 30 min, and their supernatants were used for further analysis. The time-dependent formation of methylated and fluoromethylated products, 5′-dAH, FMe- and MeCbl, as well as the decay of the carbapenam substrate and SAH were determined by high-resolution LC–MS. The detection of substrates and products was performed using electrospray ionization (ESI^+^) in positive mode, and quantification was based on standard curves of substrates and products. The assay mixture was separated on an Agilent Technologies Zorbax Extend-C18 column Rapid Resolution HT (4.6 mm × 50 mm, 1.8 μm particle size) equilibrated in 98% Solvent A (0.1% formic acid, pH 2.6) and 2% Solvent B (0.1% formic acid in 100% acetonitrile). A gradient of 2–100% Solvent B was applied from 2 to 14 min and maintained at 100% Solvent B until 17 min before returning to 2% Solvent B from 17 to 18.5 min. A flow rate of 0.3 mL/min was maintained throughout the method. The column was allowed to re-equilibrate for an additional 2.5 min under the initial conditions between sample injections.

### LC–MS Activity Assays of Fom3

Activity measurements were conducted in a Coy anaerobic chamber containing <1 ppm of O_2_. Each reaction contained the following at their final concentrations in a final volume of 270 μL:100 mM HEPES, pH 7.5, 100 mM KCl, 20 μM Fom3, 0.2 mM SAH, 10 mM FMeI, 75 μM HMT, 1.5 mM cytidylyl-2-hydroxyethylphosphonate substrate, 2 mM of TiCi, and 100 μM l-tryptophan (internal standard). Reactions were initiated by adding HMT, and 30 μL aliquots were removed at various times and added to an equal volume of 400 mM H_2_SO_4_ to quench the reaction. The samples were centrifuged at 14,000*g* for 30 min, and their supernatants were used for further analysis. The time-dependent formation of the fluoromethylated product, 5′-dAH, and FMeCbl was determined by high-resolution LC–MS. The detection of substrates and products was performed using electrospray ionization (ESI^+^) in positive mode. The assay mixture was separated on an Agilent Technologies Zorbax Extend-C18 column Rapid Resolution HT (4.6 mm × 50 mm, 1.8 μm particle size) equilibrated in 98% Solvent A (0.1% formic acid, pH 2.6) and 2% Solvent B (0.1% formic acid in 100% acetonitrile). A gradient of 2–100% Solvent B was applied from 2 to 14 min and maintained at 100% Solvent B until 17 min before returning to 2% Solvent B from 17 to 18.5 min. A flow rate of 0.3 mL/min was maintained throughout the method. The column was allowed to re-equilibrate for an additional 2.5 min under the initial conditions between sample injections.

## Results and Discussion

B_12_-RSMTs catalyze radical-dependent methylation using a ping-pong mechanism, consuming two SAM molecules in one catalytic cycle. One SAM molecule is used to methylate cob­(I)­alamin to generate MeCbl using a polar S_N_2 mechanism, while the second SAM molecule is used to generate the 5′-dA^•^ (Figure S1). In our initial attempt at radical fluoromethylation with FMeTeSAM,^25^ we found that it can effectively fluoromethylate Cbl to yield fluoromethylcobalamin (FMeCbl). However, FMeTeSAM did not produce the 5′-dA^•^ necessary to initiate the radical chemistry in any of the enzymes we tested (Figure S2). Recently, Seebeck et al. reported an enzymatic system consisting of halide methyltransferase (HMT), which uses SAH and fluoromethyl iodide (FMeI) to produce the highly unstable F-SAM molecule *in situ*.^26^ Anticipating that F-SAM could be reductively cleaved to generate a 5′-dA^•^, we coupled the HMT system with several B_12_-RSMTs, including Mmp10, CysS, and TokK to assess whether FMe groups could be transferred to their substrates.

### Fluoromethylation on a Peptide Substrate by Mmp10

Methyl coenzyme M reductase (MCR) catalyzes the last step in methane production by methanogenic archaea.^59^ The α-subunit of MCR (McrA) has several unique post-translational modifications (PTMs), including 1-*N*-methylhistidine, *S*-methylcysteine, (*S*)-δ-methylarginine, and (*S*)-α-methylglutamine.^60^ The formation of (*S*)-δ-methylarginine is catalyzed by Mmp10, a B_12_-RSMT.^61^ Radle et al. reconstituted the in vitro activity of Mmp10 from Methanosarcina acetivorans (*Ma*Mmp10) using a 13-mer peptide (^279^EMLPAR^285^ **R**ARGPNE^291^) as a substrate surrogate of McrA (Figure 2A).^49^ Mechanistic and crystallographic studies show that Mmp10 methylates C_δ_ of Arg285 using the ping-pong mechanism described in Figure S1.^62^ Given the established radical-dependent methylation of Mmp10, we developed a cascade reaction consisting of Mmp10, HMT, and the 13-mer peptide substrate.

**Figure 2:**
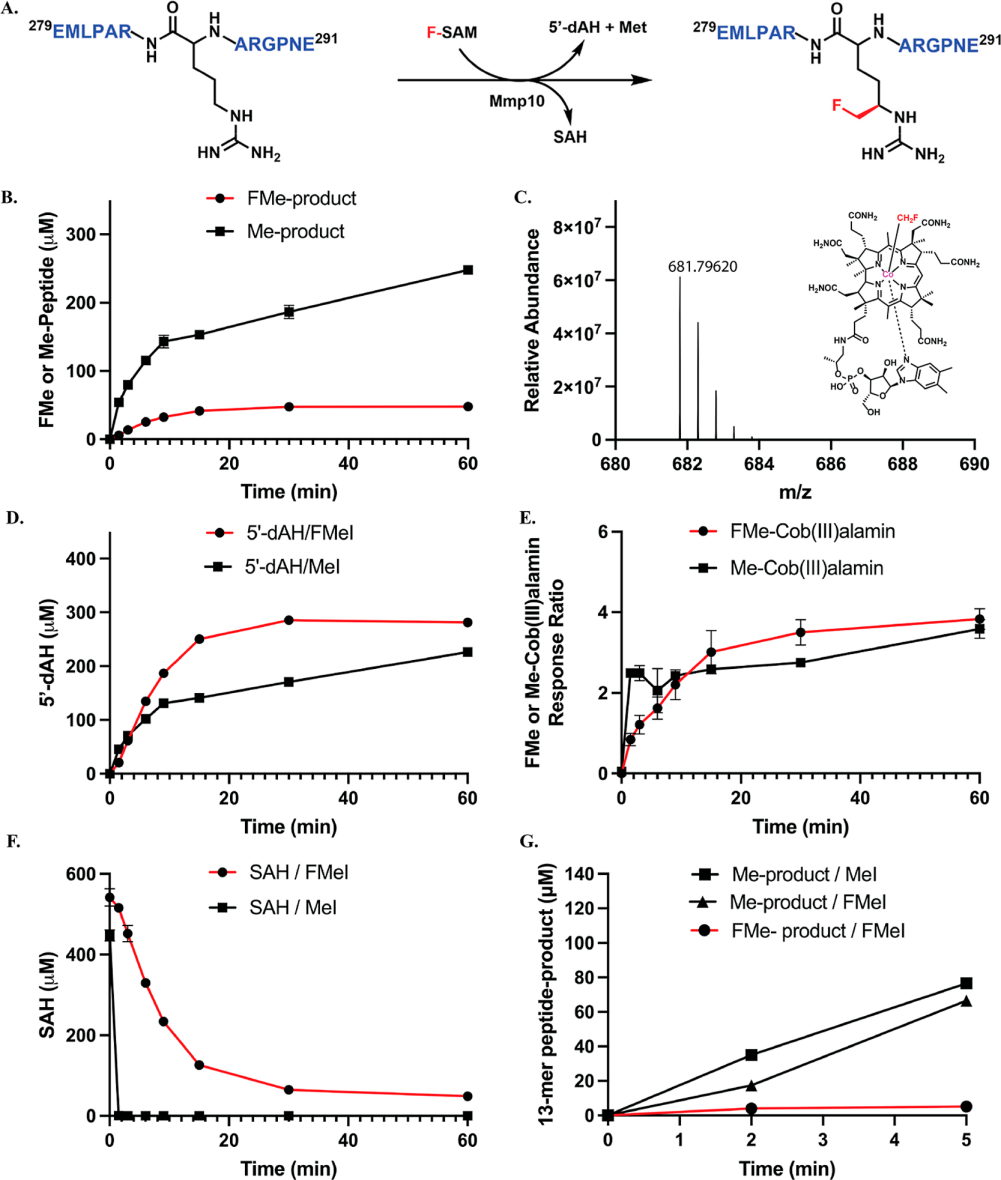
(A) Fluoromethylation catalyzed by Mmp10 on a 13-mer peptide substrate; (B) Time-dependent formation of methylated and fluoromethylated products from an SAH/MeI/Mmp10 reaction or an SAH/FMeI/Mmp10 reaction; (C) HRMS of FMeCbl generated from SAH/FMeI/Mmp10 reaction; (D) Time-dependent formation of 5′-dAH from the two reactions; (E) Time-dependent formation of MeCbl or FMeCbl from the two reactions; (F) Time-dependent decay of SAH from the two reactions; (G) Time-dependent formation of methylated and fluoromethylated products in reactions with premethylated or prefluoromethylated Cbl. The reactions contained 15 μM Mmp10, 500 μM 13-mer peptide substrate, 400 μM SAH, 100 μM HMT, 10 mM FMeI or MeI and l-tryptophan (internal standard). Reactions were run in triplicate. Error bars represent one standard deviation from the mean.

Mmp10 was overexpressed without Cbl in the cell growth media and reconstituted with OHCbl during purification to avoid contamination with MeCbl.^49,51^ Mmp10 was coupled to HMT in the presence of SAH and FMeI to generate F-SAM *in situ*. Also included were the 13-mer peptide substrate and TiCi as the reductant required to initiate the formation of the 5′-dA^•^. Similar reactions using MeI instead of FMeI were conducted in parallel as a control to compare fluoromethylation activity with methylation activity. In both reactions, the time-dependent formation of FMe- or Me-containing products is observed (Figure 2B). The formation of FMeCbl in the reaction using FMeI was confirmed by high-resolution mass spectrometry (HRMS), suggesting that radical-dependent fluoromethylation is mediated by a FMeCbl intermediate (Figure 2C, [M+2H]^2+^ calculated = 681.79647 Da, observed = 681.79620 Da, −0.4 ppm). The rate of fluoromethylation is slower than that of methylation, exhibiting ∼14 μM (∼1 equiv) of the FMe-containing product versus ∼80 μM (∼5 equiv) of the Me-containing product after three min, based on quantifying the FMe product using a Me product standard. The concentration of 5′-dAH generated in both reactions is similar, suggesting the reductive cleavage of SAM or F-SAM does not limit catalysis (Figure 2D). We do not observe the coproduct of the reductive cleavage of F-SAM, which is *S*-(fluoromethyl)- *l* -homocysteine, due to its instability.^63^ Additionally, the time-dependent formation of FMeCbl and MeCbl and the decay of SAH (substrate of HMT to generate SAM or F-SAM) are observed, as expected.

FMeI or MeI can chemically fluoromethylate or methylate cob­(I)­alamin with similar rates without HMT (Figure 2E). However, SAH decay in the SAH/FMeI/HMT coupled assay is slower than in the SAH/MeI/HMT coupled assay because FMeI is less electrophilic than MeI (Figure 2F). These data suggest that Cbl is primarily fluoromethylated or methylated chemically in the reaction and is not rate-limiting.

To identify the slow step in the reaction, the Cbl in Mmp10 was first fluoromethylated by incubating it with FMeI and 1.5 mM TiCi, followed by gel filtration to remove excess reagents. The fluoromethylated Mmp10 was reacted with SAM to generate the 5′-dA^•^ and initiate the reaction. A parallel reaction using MeI was also conducted. The results show that the rate of formation of the fluoromethylated product is much slower (∼5 μM at 5 min) in this case as well (Figure 2G). Moreover, the formation of the methylated product is much more pronounced (∼66 μM at 5 min) than the fluoromethylated product, indicating that upon donating the FMe group, the Cbl is quickly remethylated by SAM, which methylates the peptide substrate much faster than does F-SAM. These observations indicate that transferring the FMe group from FMeCbl to the peptide substrate is likely the slow step during catalysis.

### Fluoromethylation by CysS

CysS is a B_12_-RSMT involved in the biosynthesis of cystobactamids.^64^ These natural products exhibit potent antibacterial properties against the Gram-positive bacterium Staphylococcus aureus and the Gram-negative bacterium *Acinetobacter baumannii* (Figure S3A).^65,66^ CysS methylates pantothenylated 3-methoxy-4-amino benzoic acid (OMe) to produce the ethoxy (OEt) product, which it can further methylate to give *i*-propoxy (O^*i*^Pr), *s*-butoxy (O^*s*^Bu), or *t*-butoxy groups (O^*t*^Bu) (Figure S3B).^67–69^ Like Mmp10, CysS uses a Cbl cofactor to catalyze radical-dependent methylation in a similar manner (Figure S3C).

CysS was first tested for its ability to fluoromethylate the OMe-containing substrate in an SAH/FMeI/HMT coupled reaction (Figure 3A). In parallel reactions, FMeI was replaced with MeI to compare fluoromethylation and methylation activities. CysS (50 μM) was incubated with 400 μM SAH and 500 μM of the OMe-containing substrate along with 100 μM HMT and 2 mM TiCi. Time-dependent formation of the fluoromethylated (OFEt) and methylated products (OEt) is observed (Figure 3B). Although both reactions give similar amounts of products (∼55 μM after 2 h, 1 equiv to CysS concentration), the rate of fluoromethylation is slower than that of methylation (3.2 μM of OFEt product compared to 33 μM of OEt product at five min) based on the quantification of the OFEt product using the OEt product standard. The formation of 5′-dAH, FMeCbl, or MeCbl, and the decay of SAH from both reactions are similar to that observed in the Mmp10 reactions (Figure S4). Given that CysS catalyzes iterative methylations, we also observe the formation of the dimethylated (O^*i*^Pr) product in the SAH/MeI/HMT reaction, yielding ∼75 μM (1.5 equiv) of the O^*i*^Pr product (Figure 3B). However, a very low yield of difluoromethylated products is observed in the SAH/FMeI/HMT reaction, below the lowest concentration in the standard curve. Interestingly, four different species generated from the second fluoromethylation in the SAH/FMeI/HMT reaction are identified (Figure 3C). Products I and II have the same *m*/*z* as the difluoromethylated product of the OMe-containing substrate but show different retention times by liquid chromatography. We assume they are regio isomers, with the second fluoromethylation occurring on two different carbons of the OFEt group (Figure 3C). This observation suggests that H^•^ abstraction could happen on either carbon atoms of the OFEt group, likely due to a slight movement of the substrate in the active site. Product III has a vinyl group, which should derive from fluoride elimination. A methylated product IV from the OFEt-containing substrate is also detected, most likely a result of a minor contamination of FMeI with MeI. Upon closer inspection, products I and II are produced in an equal ratio. A comparison of the area of these product peaks shows that the fluoride elimination product III is produced in higher amounts than the other species (Figure S5).

**Figure 3:**
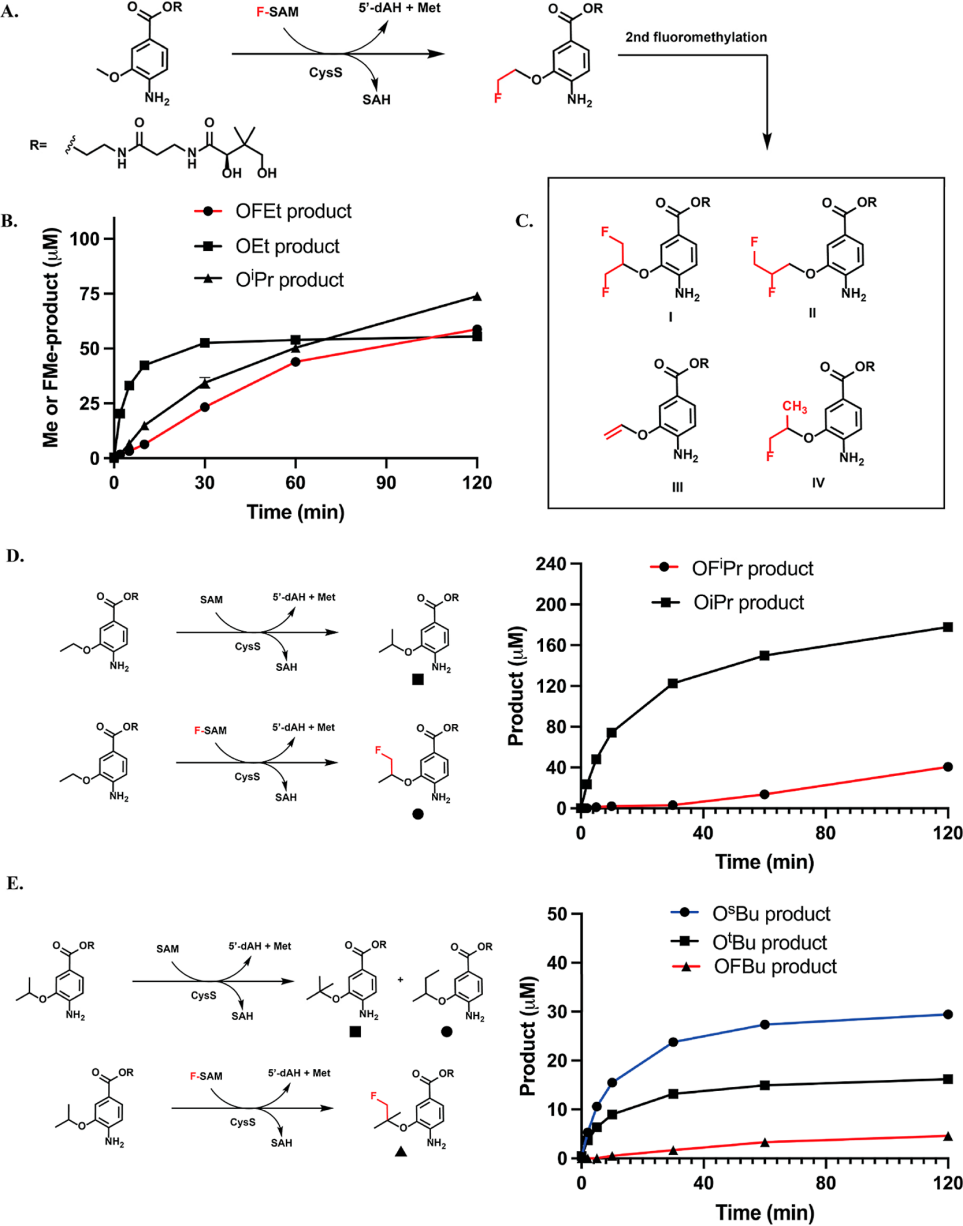
(A) The reaction catalyzed by CysS on the OMe-containing substrate; (B) time-dependent formation of methylated, dimethylated, and fluoromethylated product; (C) structures of four species observed as the result of the second fluoromethylation; (D) time-dependent formation of methylated and fluoromethylated products in the CysS reaction using OEt as the substrate; (E) time-dependent formation of methylated and fluoromethylated products in the CysS reaction using O^*i*^Pr as the substrate. The reactions contained 50 μM CysS, 500 μM substrate, 400 μM SAH, 100 μM HMT, 10 mM FMeI or MeI and l-tryptophan (internal standard). Reactions were run in triplicate. Error bars represent one standard deviation from the mean.

Given that the above observations suggest the fluorine atom in the OFEt-containing product influences the second fluoromethylation, separate reactions were conducted using OEt- and O^*i*^Pr-containing substrates to assess whether the second fluoromethylation is feasible when starting from bulkier substrates (OEt and O^*i*^Pr). With the OEt-containing substrate, the SAH/FMeI/HMT reaction gives less than one equiv of the fluoromethylated product. By contrast, the SAH/MeI/HMT reaction gives about four equiv of the methylated product (Figure 3D). The formation of 5′-dAH, FMeCbl, or MeCbl, and the decay of SAH are similar to that observed with the OMe-containing substrate (Figure S6). Interestingly, when using the O^*i*^Pr-containing substrate and SAH/MeI/HMT, two OBu-containing products (*sec*-butyl or *tert*-butyl) are detected. However, in the reaction using the O^*i*^Pr-containing substrate and SAH/FMeI/HMT, only one OFBu-containing product is detected, with a retention time closer to the *tert*-butyl product from the SAH/MeI/HMT reaction. Without authentic standards, we cannot determine whether the fluoromethylation occurs on the methyl or the methide group of the O^*i*^Pr moiety. Additionally, the formation of 5′-dAH, FMeCbl, or MeCbl, and the decay of SAH from both reactions is similar to that with the OMe- and OEt-containing substrates (Figure S7).

### Effect of Cbl Coordination Sphere on CysS Fluoromethylation

Our previous studies of TsrM and TokK suggest that the amino acid residue in the lower coordination sphere of the Cbl cofactor can influence Cbl’s reactivity. TsrM, which catalyzes a heterolytic cleavage of MeCbl, contains an Arg in that position. Substitution with other amino acids led to proteins with significantly compromised activities.^42^ By contrast, TokK, which catalyzes a homolytic cleavage of MeCbl, contains a Trp in that position. Substitution with other amino acids, except for Lys, had relatively minor effects on catalysis, and some substitutions enhanced catalysis.^43^ To try to improve the efficiency of radical fluoromethylation, we created several variants of CysS containing single substitutions to W75 on the lower face of Cbl, with the goal of impacting the strength of the Co–CH_2_F bond in enzyme-bound FMeCbl. We chose amino acids with positive (W75K, W75R), negative (W75D, W75E), and neutral (W75C, W75H) side chains (Table S1). The variants bind Cbl to varying degrees after isolation and chemical reconstitution with Cbl, iron, and sulfide (Table S2). Therefore, the variant concentrations in our assays were normalized to bound Cbl concentrations. The extent of the time-dependent formation of 5′-dAH is lower than that of WT CysS for all variants except W75H (Figure S8). Similarly, the formation of the OFEt-containing product was also significantly lower (<1 μM) for all variants except W75H. Because it was difficult to quantify the OFEt-containing product based on the standard curve using the OEt-containing standard, we used the OFEt-containing product response area (area of OFEt product/area of internal standard) to compare between WT CysS and variants. The formation of the OFEt-FMe (product of the second fluoromethylation) product is also low and below the range of the standard curve. Additionally, the time-dependent formation of FMeCbl is also low for the variants relative to the WT CysS, although the decay of the SAH substrate is observed as expected (Figure S8). SAH decay varies slightly in all variants compared to WT CysS, suggesting that HMT-catalyzed F-SAM production is not limiting among all variants.

### Fluoromethylation by TokK

TokK is a B_12_-RSMT involved in the biosynthesis of asparenomycin A, an important carbapenem antibiotic (Figure S9A).^43^ Its substrate is a pantothenylated β-lactam. Like CysS, TokK methylation proceeds up to three times sequentially, generating an isopropyl moiety as the final product (Figure S9B,C).^43,46^ Unlike CysS, TokK methylation is initiated at a methylene carbon (C6), while CysS methylation is initiated on the methyl carbon of a methoxy group. However, our studies above indicate that CysS can also fluoromethylate an OEt-containing substrate on a methylene carbon. Therefore, we tested a coupled reaction of SAH/FMeI/HMT with TokK on its carbapenam substrate (Figure 4A). Parallel reactions were conducted with MeI to compare the activity between fluoromethylation and methylation and the formation of 5′-dAH. TokK (50 μM) was incubated with 400 μM SAH and 150 μM substrate, along with 100 μM HMT and 1.5 mM TiCi. The time-dependent formation of fluoromethyl or methylated products is observed (Figure 4B). We do not have methylated/fluoromethylated product standards for the TokK reaction; therefore, we used the response ratio (area of product/area of internal standard) to compare their trends directly. The formation of the fluoromethylated product can be verified by HRMS (Figure 4C). Due to the difficulty of quantifying both products, we cannot compare the efficiency of methylation and fluoromethylation. However, according to the observation in the Mmp10 and CysS reactions, the rate of fluoromethylation should be lower than that of methylation, although this effect is minimal in the production of 5′-dAH (Figure 4D). Although TokK catalyzes iterative methylations, we do not observe significant amounts of the dimethyl or trimethyl products using the SAH/MeI/HMT coupled assay under our conditions. Thus, no further fluoromethylated products are detected in the SAH/FMeI/HMT coupled assay.

**Figure 4:**
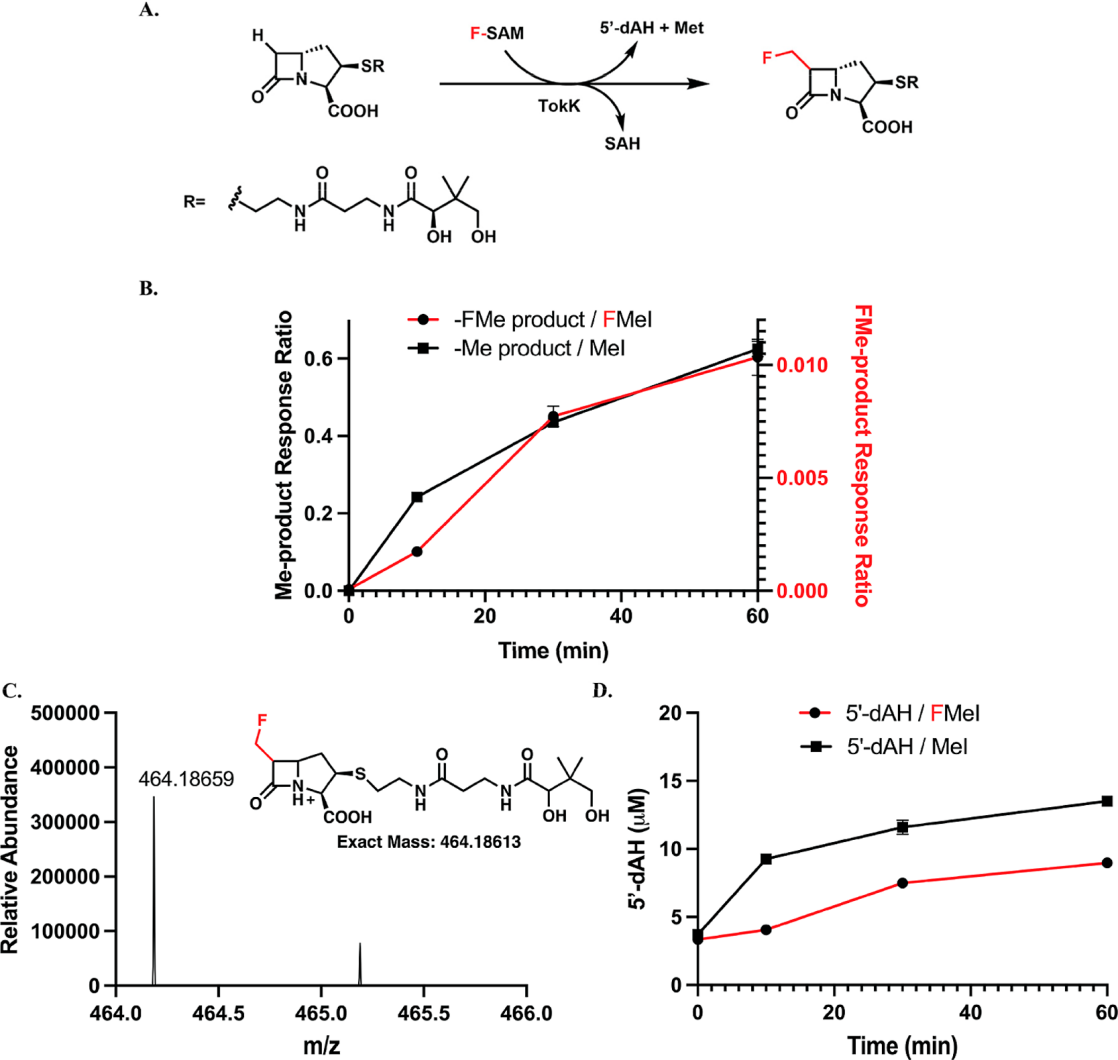
(A) Fluoromethylation catalyzed by TokK on a β-lactam substrate; (B) time-dependent formation of methylated and fluoromethylated products; (C) HRMS of fluoromethylated product from SAH/FMeI/TokK reaction; (D) time-dependent formation of 5′-dAH from the two reactions. The reactions contained 55 μM TokK, 150 μM substrate, 400 μM SAH, 100 μM HMT, 200 μM OHCbl, 10 mM FMeI or MeI, and l-tryptophan (internal standard). Reactions were run in triplicate. Error bars represent one standard deviation from the mean.

## Summary and Conclusions

In this work, we showed that transiently generated F-SAM can be used by several B_12_-RSMTs to fluoromethylate unactivated sp^3^-carbon centers. Our strategy leveraged findings by Seebeck et al., showing that F-SAM, though unstable, can be generated from SAH and FMeI using HMT. B_12_-RSMTs require 2 SAM molecules to catalyze their reactions via a ping-pong mechanism. The first SAM molecule is used to methylate cob­(I)­alamin to generate MeCbl, while the second is used to generate the 5′-dA^•^, the initiator of radical-dependent chemistry. We showed that several B_12_-RSMTs can generate the 5′-dA^•^ from F-SAM and identified the key fluoromethylating intermediate, FMeCbl, by HRMS. There were some B_12_-RSMTs that did not catalyze detectable fluoromethylation. One example is Fom3, which catalyzes the methylation of cytidylyl-2-hydroxyethylphosphonate to give cytidylyl-2-hydroxypropylphosphonate during the biosynthesis of Fosfomycin, a clinically important antibiotic (Figure S10).^58^

Our findings underscore the catalytic potential of B_12_-RSMTs with F-SAM analogs and broaden the scope of appending fluoromethyl moieties on non-nucleophilic carbon centers, expanding the ability to generate fluoromethylated natural products that may have clinical or agricultural value. While preparing this manuscript, Dong and co-workers reported a similar strategy to perform radical fluoromethylation using B_12_-RSMTs and a different set of enzymes except for CysS.^48^

## Supplementary Material



## Notes

Figures S1–S10, codon-optimized gene sequence of *cc*CysS, CysS W75 mutagenesis primers, Tables S1–S4, synthesis of CysS substrates and products, and NMR spectra of CysS substrates and products (PDF)

## Abbreviations

SAM: *S*-adenosylmethionine
SAH: *S*-adenosyl-l-homocysteine
Cbl: cobalamin
5′-dA: 5′-deoxyadenosine
FMeI: fluoromethyl iodide
FMe: fluoromethyl group
F-SAM: fluoromethyl SAM
FMeCbl: fluoromethylcobalamin
HMT: halide methyltransferase
HRMS: high-resolution mass spectroscopy
MeI: methyl iodide
MTase: methyltransferase
MeCbl: methylcobalamin
MCR: methyl coenzyme M reductase
RS: radical *S*-adenosylmethionine
RSMTs: radical *S*-adenosylmethionine methylases
OHCbl: hydroxycobalamin
PTMs: posttranslational modifications
TiCi: titanium­(III) citrate
WT: wild-type
