# Supplementary material for: Radical Fluoromethylation Enabled by Cobalamin-Dependent Radical SAM Enzymes
Source: ACS Bio Med Chem Au. 2025 May 6;5(3):464–74. doi: 10.1021/acsbiomedchemau.5c00062 (PMC12183590; doi:10.1021/acsbiomedchemau.5c00062)
Supplement: Supplementary file 1 [file bg5c00062_si_001.pdf]

Supporting Information for

Radical Fluoromethylation Enabled by Cobalamin-dependent Radical SAM Enzymes

Syam Sundar Neti[‡,#,†], Bo Wang[‡,#,†], Jiayuan Cui[‡], David F. Iwig[‡,#], Nicholas J. York[‡], Anthony J. Blaszczyk[§], Matthew R. Bauerle[‡] and Squire J. Booker*[‡,§,#]*

[‡]Department of Chemistry, The Pennsylvania State University, University Park, Pennsylvania 16802, USA.

[§]Department of Biochemistry and Molecular Biology, The Pennsylvania State University, University Park, Pennsylvania 16802, USA.

[#]Howard Hughes Medical Institute, The Pennsylvania State University, University Park, Pennsylvania 16802, USA.

† These authors contributed equally

*To whom correspondence should be addressed: Squire J. Booker (sjb14@psu.edu) or Bo Wang (bzw10@psu.edu)

Table of Contents

Page 2. Table of Contents

Page 3. Figure S1. Mechanism of arginine methylation by Mmp10

Page 3. Figure S2. FMeTeSAM generates FMeCbl but not the 5′-dA• in B_{12}-RSMT catalysis

Page 4. Figure S3. Chemical structure of cystobactamids, Iterative methylation and Catalytic mechanism of CysS

Page 5. Figure S4. Activity plots of CysS with OMe substrate

Page 6. Figure S5. Second fluoromethylation by CysS

Page 7. Figure S6. Activity plots of CysS with OEt substrate

Page 7. Figure S7. Activity plots of CysS with O^{i}Pr substrate

Page 8. Figure S8. Activity plots of CysS mutants using OMe substrate

Page 9. Figure S9. Chemical structure of Asparenomycin A, TokK reaction details and mechainsm

Page 10. Figure S10. Fluoromethylation assay with Fom3

Page 11. Codon-optimized gene sequence of *cc*CysS

Page 12. Table S1. CysS W75 mutagenic primers (5′ – 3′)

Page 13. Table S2. Quantification of cobalamin and Fe in CysS and its variants

Page 14. Table S3. HR LC-MS gradient conditions for Mmp10 product analysis

Page 14. Table S4. HR LC-MS gradient conditions for CysS and TokK product analysis

Page 15-21. Synthesis of the substrates and products of CysS

Page 22-33. NMR spectra of the substrates and products of CysS

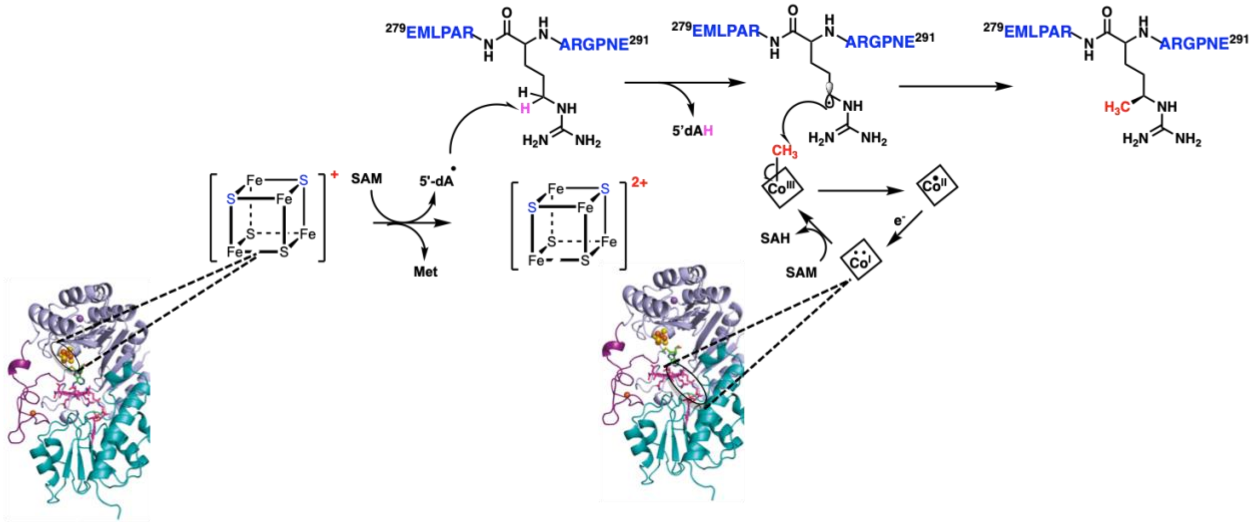

Figure S1. Mechanism of arginine methylation by *Ma*Mmp10.

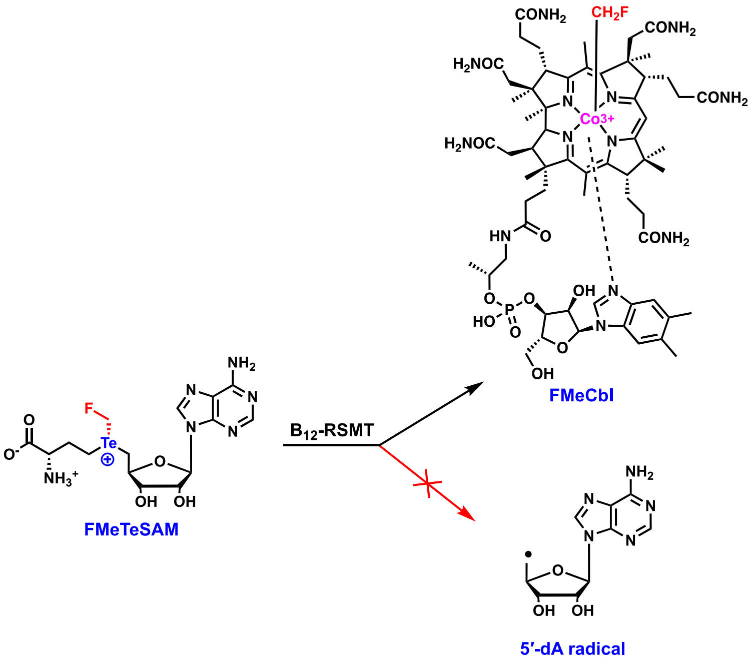

Figure S2: FMeTeSAM generates FMeCbl but not the 5′-dA• in B_{12}-RSMT catalysis.

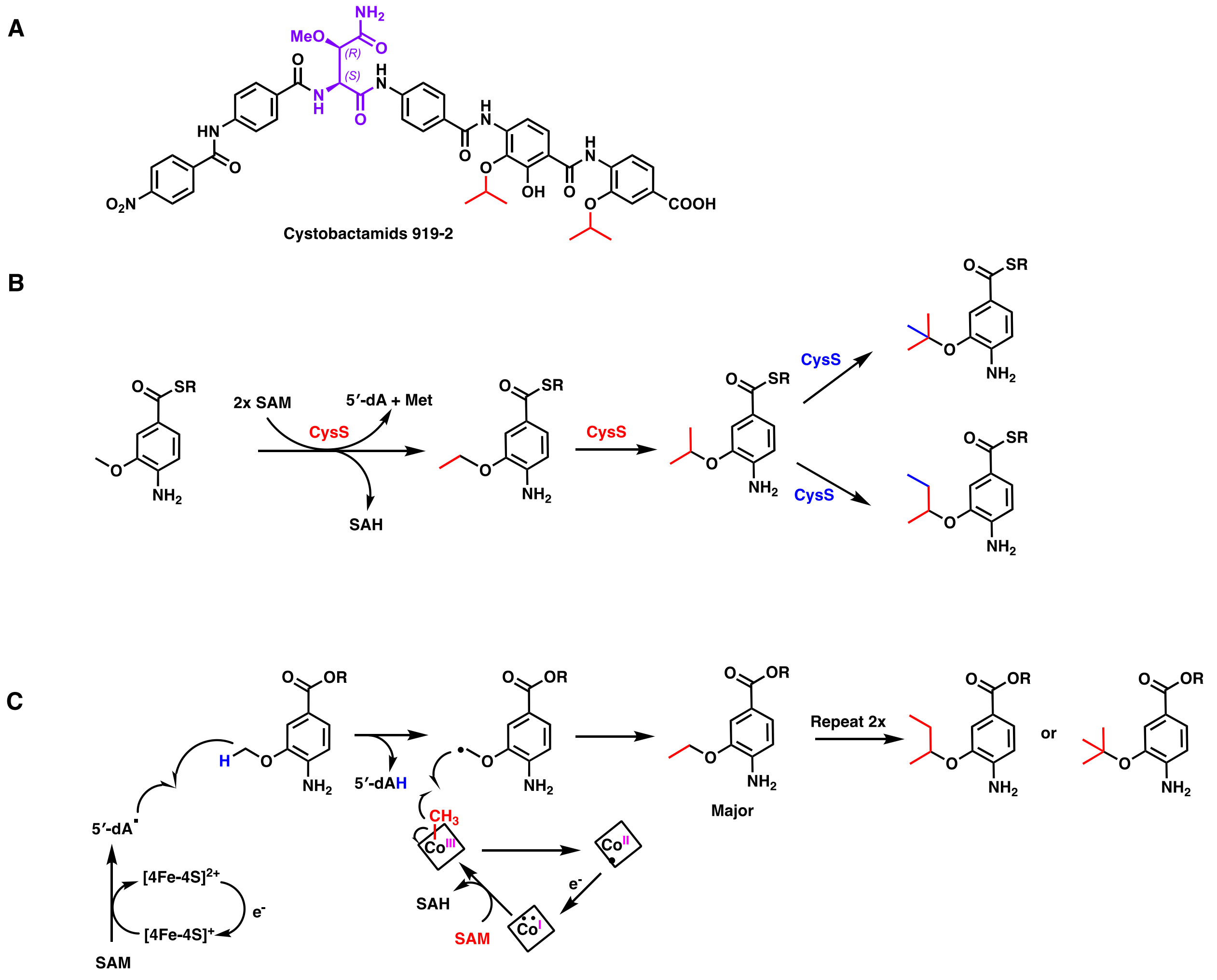

Figure S3. A) Chemical structure of cystobactamids **B)** Iterative methylation by CysS **C)** Catalytic mechanism of CysS

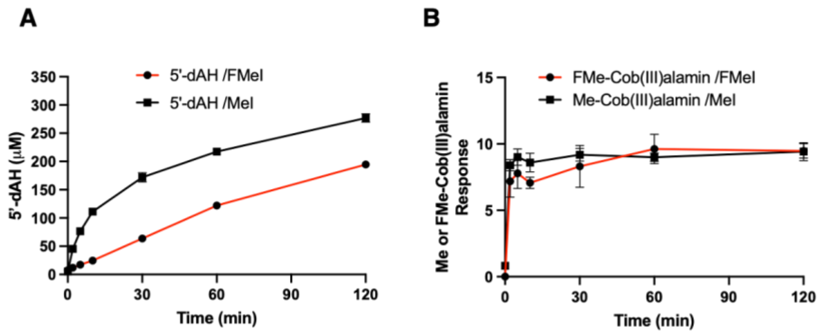

Figure S4. Time-dependent formation of **A)** 5′-dAH **B)** FMe/Me-cob(III)alamin and time-dependent decay of **C)** SAH in CysS reactions with OMe substrate. The reactions contained 50 μM CysS, 500 μM **OMe** substrate, 400 μM SAH, 100 μM HMT, 10 mM FMeI or MeI and L-tryptophan (internal standard). Reactions were initiated with MeI or FMeI and conducted at room temperature. Reactions were run in triplicate. Error bars represent one standard deviation from the mean.

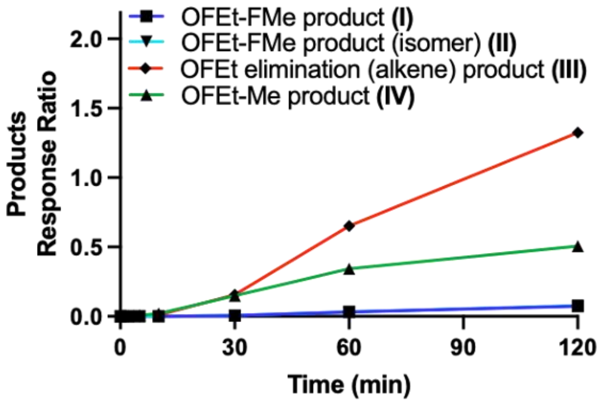

Figure S5. Comparison of CysS catalyzed second iterative fluoromethylation products response (area of the peak/area of the internal standard). -OFEt-FMe products (regioisomers, blue and cyan, I and II), -OFEt-elimination (alkene) product (red, III) and -OFEt-Me product (green, IV). The reactions contained 50 µM CysS, 500 µM **OMe** substrate, 400 µM SAH, 100 µM HMT, 10 mM FMeI and L-tryptophan (internal standard). Reactions were run in triplicate. Error bars represent one standard deviation from the mean.

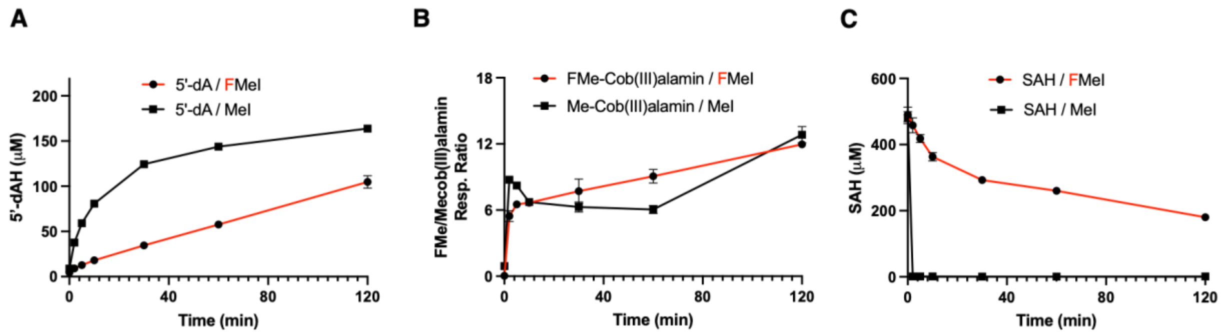

Figure S6. Time-dependent formation of **A)** 5′-dAH **B)** FMe/Me-cob(III)alamin and time-dependent decay of **C)** SAH in CysS reactions with **OEt** substrate. The reactions contained 50 μM CysS, 500 μM **OEt** substrate, 400 μM SAH, 100 μM HMT, 10 mM FMeI or MeI and L-tryptophan (internal standard). Reactions were initiated with MeI or FMeI and conducted at room temperature. Reactions were run in triplicate. Error bars represent one standard deviation from the mean.

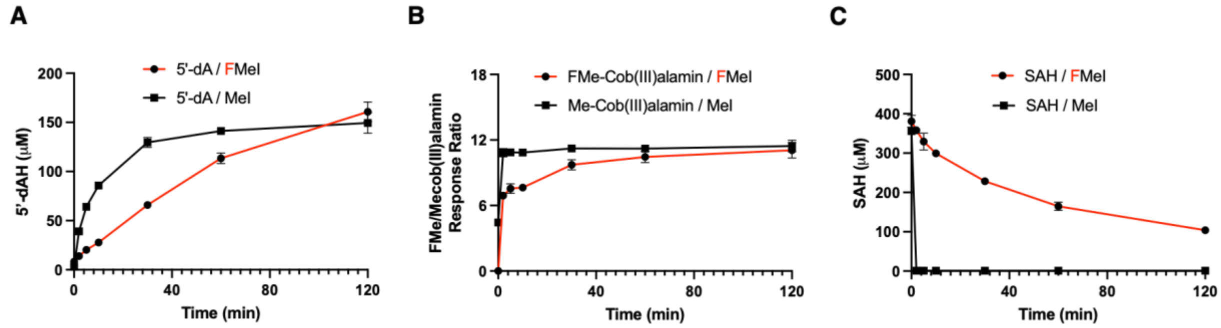

Figure S7. Time-dependent formation of **A)** 5′-dAH **B)** FMe/Me-cob(III)alamin and time-dependent decay of **C)** SAH in CysS reactions with **O*ⁱ*Pr** substrate. The reactions contained 50 μM CysS, 500 μM **O*ⁱ*Pr** substrate, 400 μM SAH, 100 μM HMT, 10 mM FMeI or MeI and L-tryptophan (internal standard). Reactions were initiated with MeI or FMeI and conducted at room temperature. Reactions were run in triplicate. Error bars represent one standard deviation from the mean.

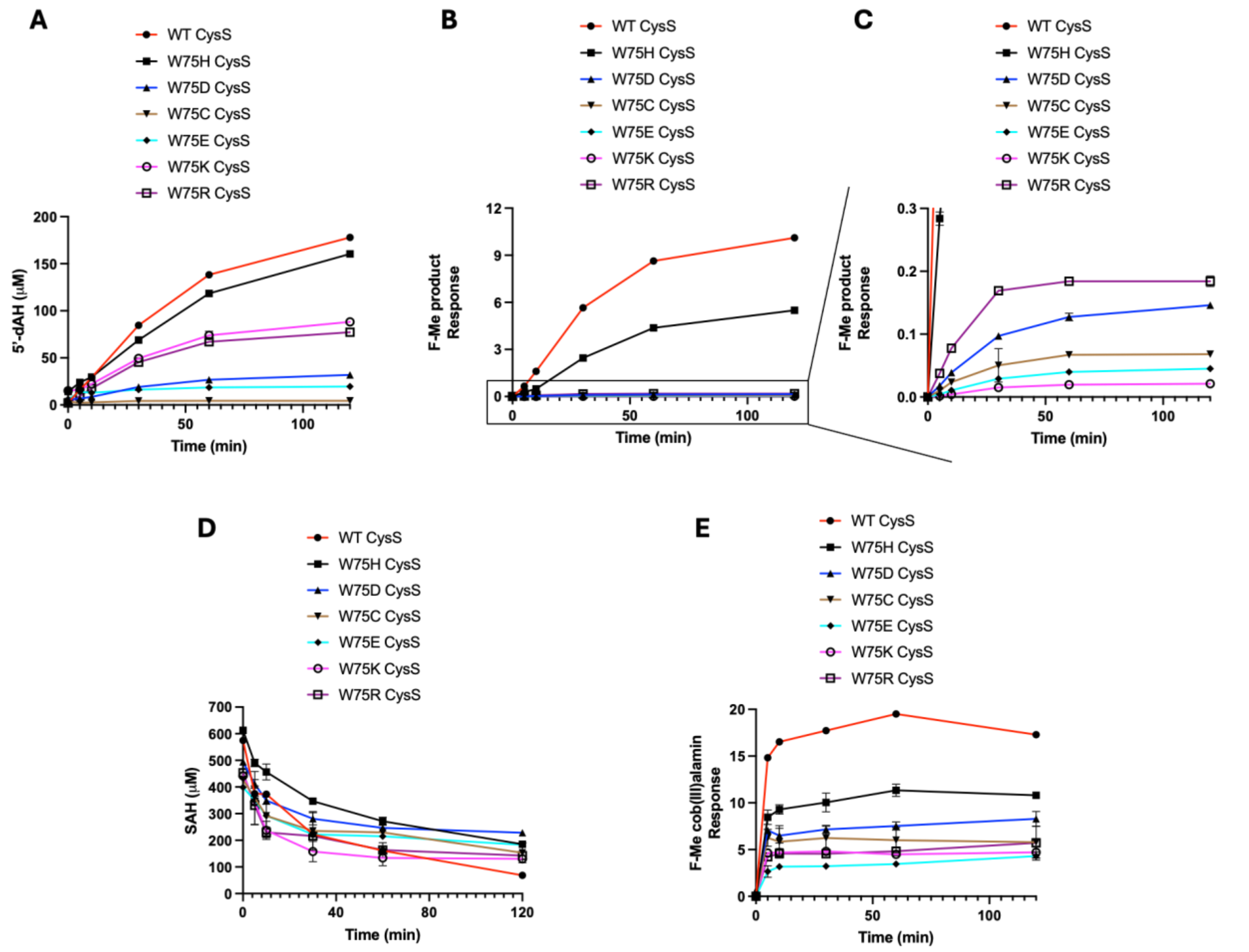

Figure S8. Coupled assay reactions of CysS variants (W75H, W75D, W75C, W75E, W75K, W75R) with SAH/HMT/FMeI. Time-dependent formation of **A)** 5′-dAH **B)** fluoromethylated product **C)** Zoomed version of **(B)**, **D)** decay of SAH **E)** formation of FMe-Cob(III)alamin. All the reactions contained normalized concentrations of 50 μM cobalamin bound CysS, 500 μM -**OMe** substrate, 2 mM Titanium citrate, 400 μM SAH, 100 μM HMT, 10 mM FMeI and L-Tryptophan (internal standard). Reactions were initiated with FMeI and conducted at room temperature. Reactions were run in triplicate. Error bars represent one standard deviation from the mean.

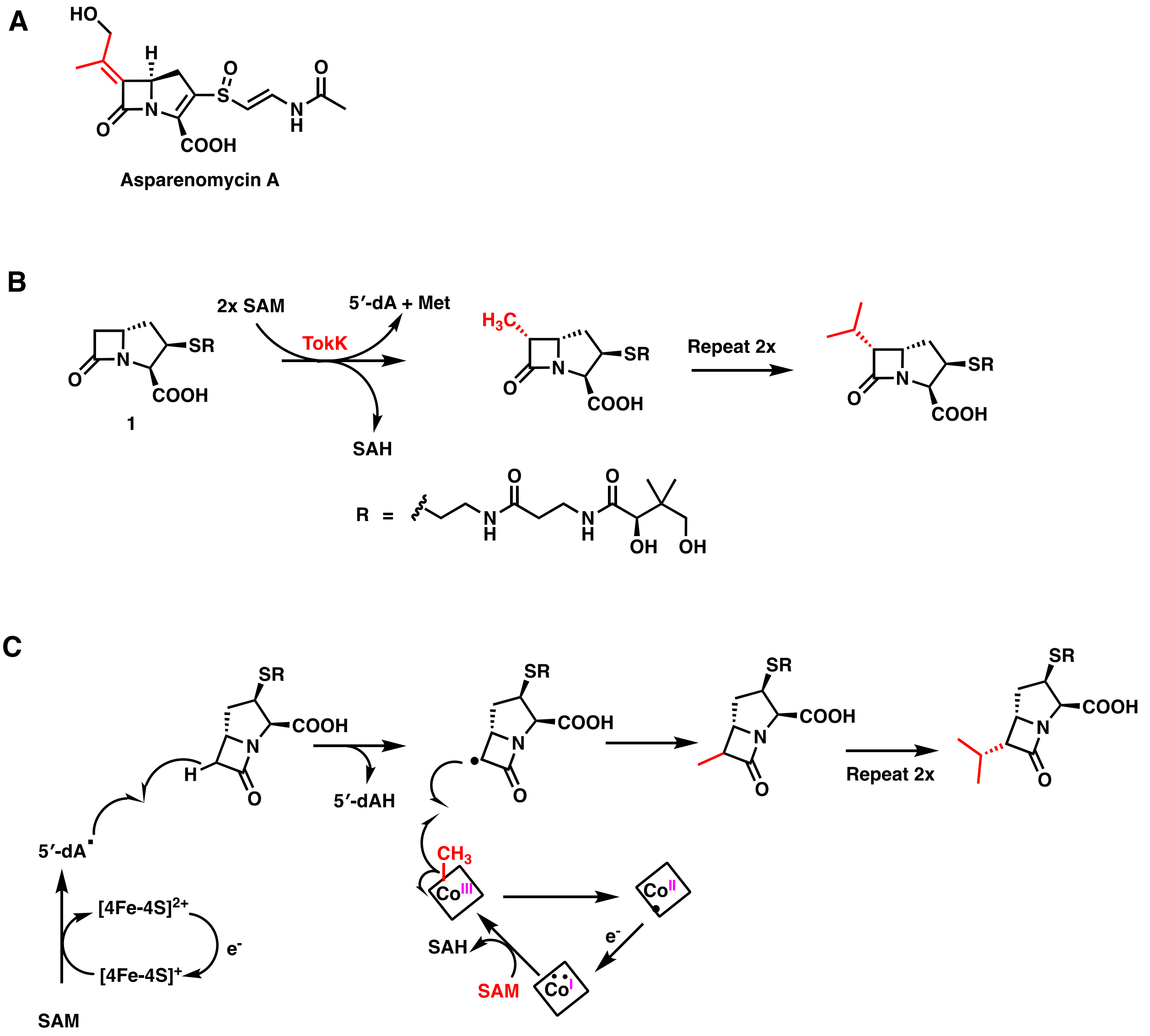

Figure S9. A) Chemical structure of Asparenomycin A **B)** Iterative methylation by TokK **C)** Catalytic mechanism of TokK.

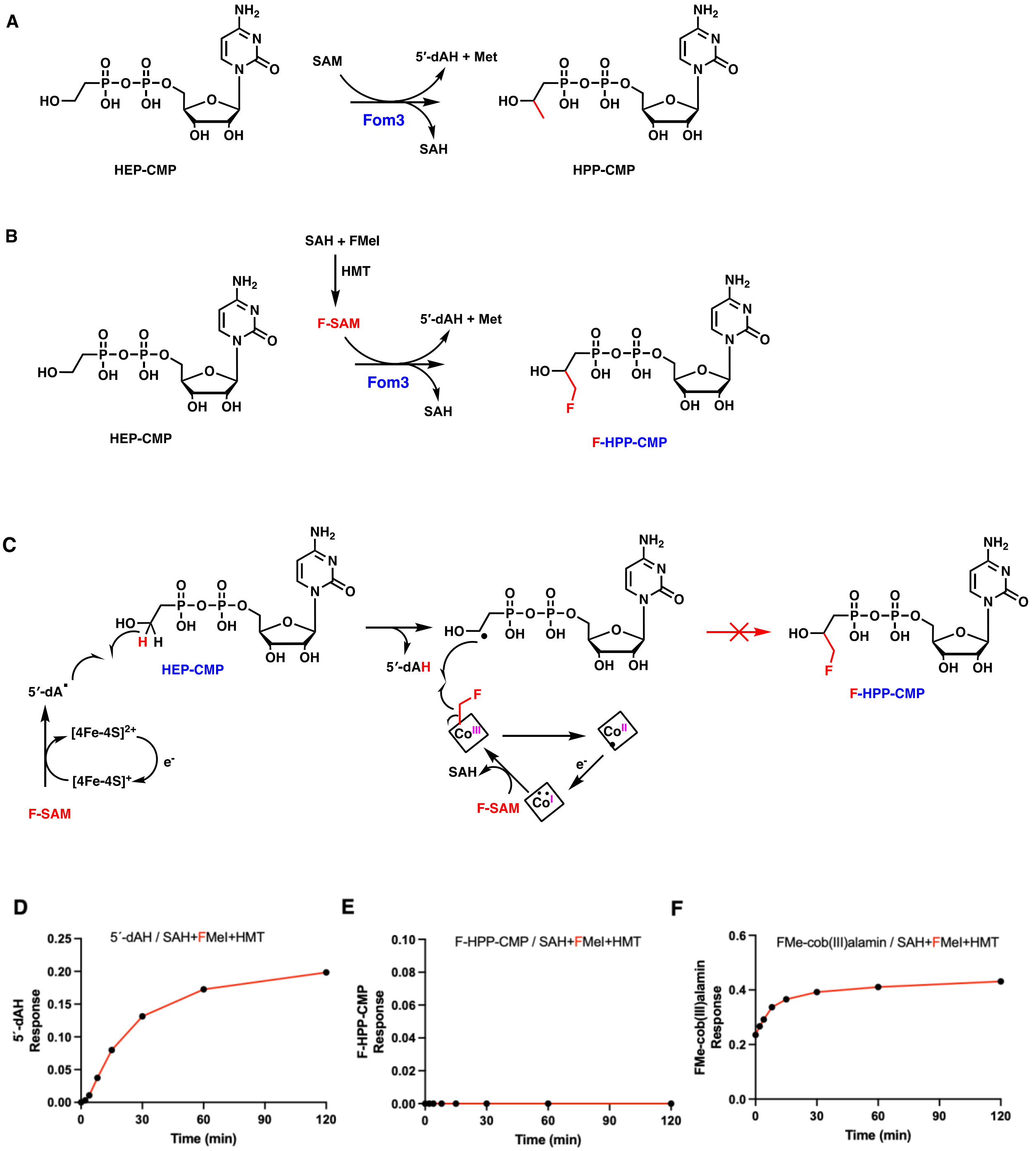

Figure S10. Schematic of **A)** Methylation **B)** Fluoromethylation **C)** Mechanism of Fluoromethylation catalyzed by Fom3. Formation of **D)** 5′-dAH **E)** F-HPP-CMP product **F)** FMe-cob(III)alamin in SAH/FMeI/HMT Fom3 assay.

Sequence of the codon-optimized gene of the *cc*CysS (UniProt ID: A0A3A8HCN5) as supplied by Gene Universal:

5′-
CATATGGGTGCAATGGTTAATCAGCGTGTTGCATTTATTGAACTGACCGTTTTGCCG
GTGTTTATCCGCTGGCAAGCGGTTATATGCGTGGTGTTGCAGAACAGAATGCAGCAA
TTAAAGATGCCTGCAGCTTTGAAATTCATAGCATCTGCATTAACGACAACCGTTTTG
AAGATCGTCTGAATGCAATTGATGCAGATGTTTATGCCATTAGCTGCTATGTTTGGA
ATATGGGTTTTGTGAAACGTTGGCTGCCGACACTGACCGCACGTAAACCGCATGCAC
ATGTTATTTTAGGTGGTCCGCAGGTTATGAATCATGGTGCACGTTATCTGGATCCGG
GTAATGAACGTGTTGTTCTGTGTAATGGTGAAGGCGAATATACCTTTGCAAATTATC
TGGCGGAAATTTGTAGTCCGGAACCGGATCTGGGTAAAGTTAAAGGCCTGACCTTTT
ATCGCAATGGTGAACTGATTACCAGCGCACCGCAAGAACGTATTCAGGATCTGAAT
GCCATTCCGAGTCCGTATCTGGAAGGTTATTTTGATAGCGAGAAATATGTTTGGGCA
CCGATTGAAACCAATCGTGGTTGTCCGTATCAGTGTACCTATTGTTTTTGGGGTGCA
GCAACCAATAGCCGTGTGTTTAAAACCGATATGGATCGTGTTAAAGCCGAAATTACC
TGGCTGAGCCAGCGTCGTGCCTTTTATATCTTTATTACCGATGCCAATTTTGGCATGC
TGACCCGTGATATTGAAATTGCACAGCATATTGCCGAATGCAAACGCAAATATGGCT
ATCCGCTGACCGTGTGGCTGAGTGCAGCCAAAAATAGTCCGGATCGTGTGACCCAG
ATTACCCGTATTCTGAGCCAAGAAGGTCTGATTAGCACCCAGCCGGTTAGCCTGCAG
ACCATGGATGCAAATACCCTGAAAAGCGTTAAACGCGGTAACATTAAAGAAAGCGC
ATATCTGAATCTGCAAGAAGAACTGCGTCGTAGCAAACTGAGCAGCTTTGTTGAAAT
GATTTGGCCGTTACCGGGTGAAACCCTGGAAACCTTTAAAGAGGGTATTGGTAAACT
GTGTAGCTATGAAGCAGATGCCATTCTGATTCATCATCTGCTGCTGATTAATAACGT
TCCGATGAATGCACAGCGCGAAGAATTTAATCTGGAAGTGAGCAATGATGAAGATC
CGAATAGCGAAGCACAGGTTGTTGTTGCAACCCGTGATGTTACCCGTGAAGAATAC
AAAGAAGGTGTGCGTTTTGGTTATCATCTGACCAGCCTGTATAGTCTGCGTGCACTG
CAGTTTGTTGGTAAATATCTGGATAAACAGGGTCTGCTGGCATTCAAAGATCTGATT
AGTAGCTTTAGCGATTACTGCAAACGTTTTCCGGATCATCCGTATACACAGTATATT
AGCAGCATTATTGATGGTAGCAGCCAGAGCAAATTTAGCGCAAATGGTGGTATTTTT
CATGTGACCCTGCATGAATTTCGTCGCGAATTTGATCAGCTGCTGGCAGGTTTTCTGC
AGAGCCTGGGTATGATGCATACCGAACCGCTGGAATTTCTGTTTGATCTGGATCTGC
TGAATCGTCCGCATGTTTATAGCAATACACCGGTTACCAATGGTGATGGTCTGCTGA
AACATGTTACCGTTGTTGCCAAAGAAAAAGATGCACTGGTTGTTCATATCCCGGAAA
AATATGTTCAGCTGGCATGGGAAATGCTGCGTCTGGATGGTGCACCGAGCACACGT
ATGCGTGTTAAATATCGTGGTGCACAGATGCCGTTTATGGCAAATAAACCGTATGAA
GATAACCTGAGCTATTGCGAAGCAAAACTGCATAAAATGGGTAGCATTCTGCCGGTT
TGGGAACCTGCAGTTCCGAGCATTGCACCGGTTCGTCGTCCTCAGGTTGCAGTTGCA
AGCTAACTCGAG-3′

CATATG – *NdeI* restriction site
CTCGAG – *XhoI* restriction site
TAA – Stop codon

Table S1: Primers for constructing CysS W75 variants (ligand underneath cobalamin)

Mutant	Forward primer	Reverse primer
W75C	5'- GCAATTAGCTGTTATGTG TGC AATATGGGTTTTGTTAAACG -3'	5'- CGTTTAACAAAACCCATATTGCACACAT AACAGCTAATTGC -3'
W75R	5'- GCAATTAGCTGTTATGTG CGT AATATGGGTTTTGTTAAACG -3'	5'- CGTTTAACAAAACCCATATTACGCACAT AACAGCTAATTGC-3'
W75K	5'- GCAATTAGCTGTTATGTG AAA AATATGGGTTTTGTTAAACG -3'	5'- CGTTTAACAAAACCCATATTTTTCACAT AACAGCTAATTGC -3'
W75D	5'- GCAATTAGCTGTTATGTG GAT AATATGGGTTTTGTTAAACG -3'	5'- CGTTTAACAAAACCCATATTATCCACAT AACAGCTAATTGC -3'
W75E	5'- GCAATTAGCTGTTATGTG GAA AATATGGGTTTTGTTAAACG -3'	5'- CGTTTAACAAAACCCATATTTTCCACAT AACAGCTAATTGC -3'
W75H	5'- GCAATTAGCTGTTATGTG CAT AATATGGGTTTTGTTAAACG -3'	5'- CGTTTAACAAAACCCATATTATGCACAT AACAGCTAATTGC -3'

Table S2: Quantification of cobalamin and Fe analysis of WT CysS and its variants

Protein	% Cobalamin in the protein	Number of Fe/protein monomer
WT CysS	94 %	3.3
W75H CysS	82 %	3.11
W75C CysS	74 %	3.83
W75D CysS	42 %	3.47
W75E CysS	64 %	3.02
W75R CysS	80 %	3.4
W75K CysS	98 %	3.42

Table S3: LC-MS gradient conditions for the analysis of substrates/products in Mmp10 reactions

Time (min)	0.1% Formic acid in H_2O	0.1% Formic acid in Acetonitrile	Flow (mL/min)
0	98%	2%	0.3
2	98%	2%	0.3
10	0%	100%	0.3
12	0%	100%	0.3
13.5	98%	2%	0.3
15.5	98%	2%	0.3

Table S4: LC-MS gradient conditions for the analysis of substrates/products in CysS/TokK reactions

Time (min)	0.1% Formic acid in H_2O	0.1% Formic acid in Acetonitrile	Flow (mL/min)
0	98%	2%	0.3
2	98%	2%	0.3
14	0%	100%	0.3
17	0%	100%	0.3
18.5	98%	2%	0.3
21	98%	2%	0.3

Synthesis of substrates and products of CysS

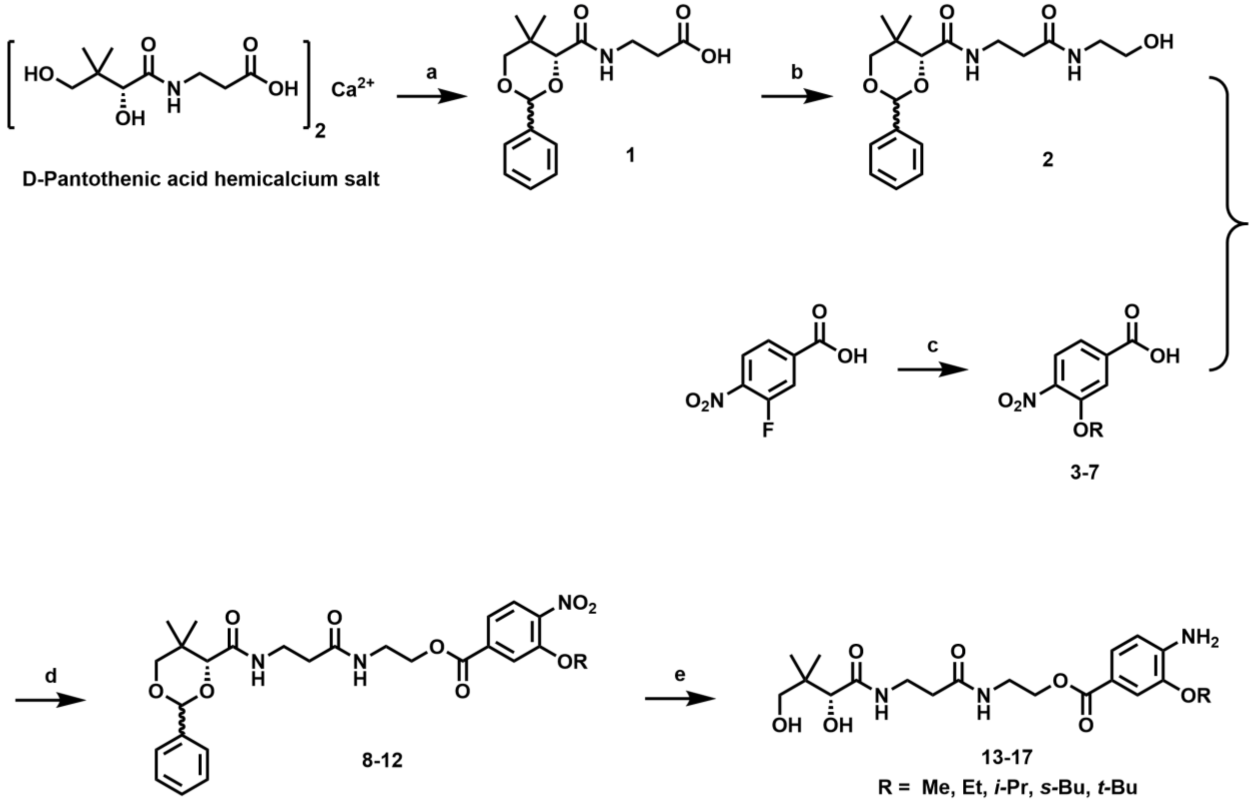

Conditions and reagents: a) benzaldehyde dimethyl acetal, *p*-TsOH, DMF, 60 ^{o}C, overnight, 55%; b) ethanolamine, HATU, NMM, DMF, rt, overnight, 65%; c) alcohol, NaH, 70 ^{o}C, overnight; d) EDC, DMAP, DCM, rt, overnight; e) Pd/C, MeOH, formic acid, H_2, 250 psi.

5.1 Synthesis of Compound 2

To a stirred solution of D-Pantothenic acid hemicalcium salt (11.9 g, 50.0 mmol, 1.0 equiv) and benzaldehyde dimethyl acetal (15.2 g, 100.0 mmol, 2.0 equiv) in DMF (100 mL) was added *p*-TsOH (9.5 g, 50.0 mmol, 1.0 equiv), and the resulting reaction mixture was stirred at 60 ^{o}C overnight. After completion, the reaction mixture was diluted with ethyl acetate (400 mL) and washed thoroughly with water and brine. The organic layer was dried over anhydrous sodium sulfate and concentrated *in vacuo*, and the resulting residue was crystallized from ethyl acetate, giving 8.4 g of compound **1** as white crystal in a yield of 55%. ^{1}H NMR (500 MHz, DMSO) δ

12.26 (s, 1H), 7.59 – 7.46 (m, 3H), 7.45 – 7.35 (m, 3H), 5.59 (s, 1H), 4.13 (s, 1H), 3.66 (q, *J* = 11.1 Hz, 2H), 3.46 – 3.32 (m, 1H), 3.32 – 3.20 (m, 1H), 2.41 (t, *J* = 6.9 Hz, 2H), 1.02 (s, 3H), 0.96 (s, 3H); ^{13}C NMR (126 MHz, DMSO) δ 173.58, 168.61, 138.52, 129.31, 128.53, 126.85, 100.90, 83.66, 77.88, 34.73, 34.22, 33.08, 22.00, 19.46.

To an ice-water cooled solution of compound **1** (6.6 g, 21.5 mmol, 1.0 equiv), ethanolamine (2.6 g, 43.0 mmol, 2.0 equiv), and N-methylmorpholine (6.5 g, 64.5 mmol, 3.0 equiv) in DMF (100 mL) was added HATU (10.6 g, 28.0 mmol, 1.3 equiv), and the resulting reaction mixture was stirred at rt overnight. After completion, the reaction mixture was diluted with ethyl acetate (400 mL) and washed thoroughly with 0.1 M HCl, saturated aqueous sodium bicarbonate, water, and brine. The organic layer was dried over anhydrous sodium sulfate and concentrated *in vacuo*, and the resulting residue was crystallized from ethyl acetate, giving 4.9 g of compound **2** as a white solid in a yield of 65%. ^{1}H NMR (500 MHz, DMSO) δ 7.88 (s, 1H), 7.58 – 7.45 (m, 3H), 7.45 – 7.34 (m, 3H), 5.59 (s, 1H), 4.63 (t, *J* = 5.5 Hz, 1H), 4.11 (s, 1H), 3.66 (q, *J* = 11.1 Hz, 2H), 3.45 – 3.30 (m, 4H), 3.29 – 3.21 (m, 1H), 3.09 (q, *J* = 5.9 Hz, 2H), 2.28 (t, *J* = 6.9 Hz, 2H), 1.01 (s, 3H), 0.96 (s, 3H); ^{13}C NMR (126 MHz, DMSO) δ 171.07, 168.51, 138.52, 129.30, 128.52, 126.83, 100.82, 83.65, 77.87, 60.30, 41.85, 35.36, 35.27, 33.07, 22.00, 19.48.

5.2 Synthesis of benzoic acid analogs 3-7

To ice-water cooled methanol (100 mL), ethanol (100 mL), isopropanol (100 mL), 2-butanol (100 mL), or *t*-butanol (100 mL) was added NaH (4.3 g, 108.1 mmol, 4.0 equiv, 60% in mineral oil) slowly in small portions. After addition, 3-fluoro-4-nitrobenzoic acid (5.0 g, 27.0 mmol, 1.0 equiv) was added to the resulting solution. The resulting reaction mixture was stirred at 70 ^{o}C overnight. After completion, the reaction mixture was concentrated *in vacuo*. The resulting residues were dissolved in ethyl acetate (400 mL) and washed thoroughly with 0.1 M HCl, water,

and brine. The organic layer was dried over anhydrous sodium sulfate and concentrated *in vacuo*, and the resulting residue was crystallized from ethyl acetate.

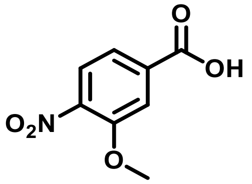

3

Yield: 85%. Pale yellow needle crystal. ^{1}H NMR (500 MHz, DMSO) δ 13.64 (s, 1H), 7.95 (d, J = 8.3 Hz, 1H), 7.76 (s, 1H), 7.64 (d, J = 8.3 Hz, 1H), 3.98 (s, 3H); ^{13}C NMR (126 MHz, DMSO) δ 166.24, 151.98, 142.38, 136.18, 125.46, 121.76, 115.01, 57.24.

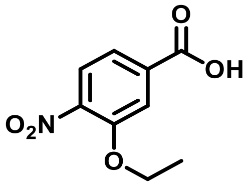

4

Yield: 90%. Yellow needle crystal. ^{1}H NMR (500 MHz, DMSO) δ 13.61 (s, 1H), 7.94 (d, J = 8.3 Hz, 1H), 7.73 (s, 1H), 7.62 (d, J = 8.3 Hz, 1H), 4.46 – 4.20 (m, 2H), 1.34 (t, J = 6.9 Hz, 3H); ^{13}C NMR (126 MHz, DMSO) δ 166.26, 151.16, 142.66, 136.06, 125.36, 121.68, 115.74, 65.78, 14.73.

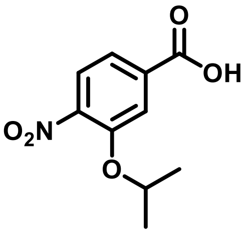

5

Yield: 90%. Yellow solid. ^{1}H NMR (500 MHz, $CDCl_3$) δ 7.79 (dd, J = 26.6, 11.1 Hz, 3H), 4.81 (dt, J = 11.7, 5.8 Hz, 1H), 1.45 (d, J = 5.7 Hz, 6H); ^{13}C NMR (126 MHz, $CDCl_3$) δ 170.37, 150.80, 144.42, 133.30, 125.18, 121.80, 117.35, 73.17, 21.78.

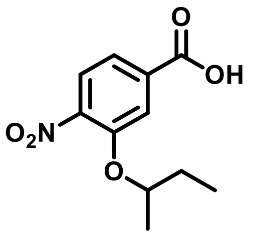

6

Yield: 75%. Yellow solid. ^{1}H NMR (500 MHz, DMSO) δ 13.60 (s, 1H), 7.92 (d, J = 8.2 Hz, 1H), 7.75 (s, 1H), 7.60 (d, J = 8.3 Hz, 1H), 4.84 – 4.62 (m, 1H), 1.76 – 1.55 (m, 2H), 1.26 (d, J = 5.9 Hz, 3H), 0.91 (t, J = 7.3 Hz, 3H); ^{13}C NMR (126 MHz, DMSO) δ 166.29, 150.31, 143.66, 135.86, 125.34, 121.60, 116.73, 77.42, 28.84, 19.12, 9.60.

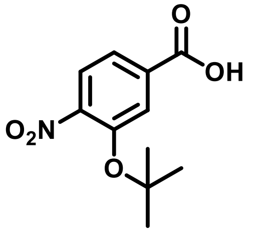

7

Yield: 70%. Yellow solid. ^{1}H NMR (500 MHz, DMSO) δ 13.62 (s, 1H), 7.92 (d, J = 8.3 Hz, 1H), 7.79 (s, 1H), 7.75 (d, J = 8.3 Hz, 1H), 1.38 (s, 9H); ^{13}C NMR (126 MHz, DMSO) δ 166.13, 148.10, 147.96, 135.17, 125.05, 124.49, 124.28, 83.37, 28.82.

5.3 Synthesis of product 13-17

To an ice-water cooled solution of compound **2** (350 mg, 1.0 mmol, 1.0 equiv) and benzoic acid analog (1.5 mmol, 1.5 equiv) in DCM (50 mL) was added EDC (233 mg, 1.5 mmol, 1.5 equiv)

and DMAP (25 mg, 0.2 mmol, 0.2 equiv), and the resulting reaction mixture was stirred at rt overnight. After completion, the reaction mixture was diluted with DCM (100 mL) and washed thoroughly with 0.1 M HCl, saturated aqueous sodium bicarbonate, water, and brine. The organic layer was dried over anhydrous sodium sulfate and concentrated *in vacuo*, and the resulting residue was purified by silica gel flash chromatography (hexanes : acetone = 2:1), giving intermediate **8-12**.

The solution of intermediate **8-12**, Pd/C (50 mg), and formic acid (50 μL) in methanol (50 mL) was hydrogenated under H2 atmosphere at 250 psi overnight. After completion, the reaction mixture was filtered to remove Pd/C. The filtrate was concentrated *in vacuo*, and the resulting residue was purified by silica gel flash chromatography (DCM: methanol = 10:1), giving final products **13-17**.

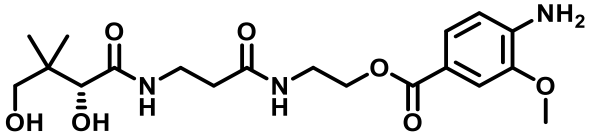

13

Yield: 65% over two steps. Yellowish syrup. ^{1}H NMR (500 MHz, D_2O) δ 8.17 (s, 1H), 7.82 (s, 1H), 7.43 (d, *J* = 8.8 Hz, 1H), 7.35 (s, 1H), 6.77 (d, *J* = 8.7 Hz, 1H), 4.36 – 4.10 (m, 1H), 3.79 (s, 3H), 3.77 (s, 1H), 3.55 – 3.43 (m, 2H), 3.42 – 3.34 (m, 2H), 3.31 (d, *J* = 11.3 Hz, 1H), 3.19 (d, *J* = 11.3 Hz, 1H), 2.38 (t, *J* = 6.8 Hz, 2H), 0.71 (s, 3H), 0.66 (s, 3H); ^{13}C NMR (126 MHz, D_2O) δ 175.08, 174.17, 168.59, 146.87, 141.34, 124.42, 119.17, 114.89, 111.88, 75.75, 68.46, 63.64, 55.86, 38.46, 35.54, 35.37, 20.35, 18.89; **HRMS**: calculated for $C_{19}H_{30}N_3O_7^+$ [M+H$^+$]: 412.20783; found: 412.20820.

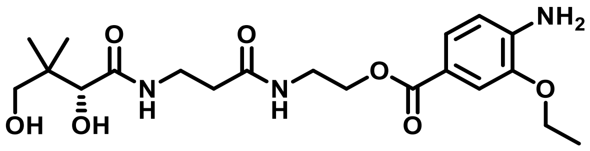

14

Yield: 70% over two steps. White foam. ^{1}H NMR (500 MHz, DMSO) δ 8.10 (t, *J* = 5.5 Hz, 1H), 7.69 (t, *J* = 5.8 Hz, 1H), 7.40 (dd, *J* = 8.2, 1.1 Hz, 1H), 7.30 (s, 1H), 6.64 (d, *J* = 8.2 Hz, 1H), 5.57 (s, 2H), 5.34 (d, *J* = 5.4 Hz, 1H), 4.45 (t, *J* = 5.5 Hz, 1H), 4.13 (t, *J* = 5.6 Hz, 2H), 4.04 (q, *J* = 6.9 Hz, 2H), 3.69 (d, *J* = 5.5 Hz, 1H), 3.44 – 3.20 (m, 7H), 3.16 (dd, *J* = 10.4, 5.3 Hz, 1H), 2.34 – 2.24 (m, 2H), 1.36 (t, *J* = 6.9 Hz, 3H), 0.79 (s, 3H), 0.77 (s, 3H); ^{13}C NMR (126 MHz, DMSO) δ 173.30, 171.24, 166.39, 144.71, 143.89, 124.60, 116.56, 112.51, 112.48, 75.46, 68.50, 64.00, 63.09, 38.20, 35.59, 35.25, 21.37, 20.76, 15.18; **HRMS**: calculated for $C_{20}H_{32}N_3O_7^+$ [M+H$^+$]: 426.22348; found: 426.22355.

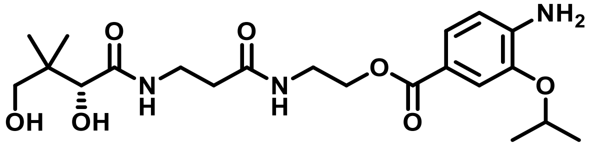

15

Yield: 70% over two steps. White foam. ^{1}H NMR (500 MHz, DMSO) δ 8.21 – 8.04 (m, 1H), 7.79 – 7.64 (m, 1H), 7.40 (d, *J* = 8.2 Hz, 1H), 7.33 (s, 1H), 6.65 (d, *J* = 8.2 Hz, 1H), 5.54 (s, 2H), 5.36 (d, *J* = 5.2 Hz, 1H), 4.58 – 4.49 (m, 1H), 4.46 (t, *J* = 5.1 Hz, 1H), 4.14 (t, *J* = 5.0 Hz, 2H), 3.70 (d, *J* = 5.2 Hz, 1H), 3.46 – 3.20 (m, 7H), 3.19 – 3.14 (m, 1H), 2.29 (t, *J* = 6.8 Hz, 2H), 1.28 (d, *J* = 5.7 Hz, 6H), 0.80 (s, 3H), 0.77 (s, 3H); ^{13}C NMR (126 MHz, DMSO) δ 173.30, 171.22, 166.36, 145.00, 143.31, 124.67, 116.59, 114.94, 112.88, 75.44, 70.93, 68.49, 63.09, 38.20, 35.59, 35.24, 22.32, 21.37, 20.75; **HRMS**: calculated for $C_{21}H_{34}N_3O_7^+$ [M+H$^+$]: 440.23913; found: 440.23955.

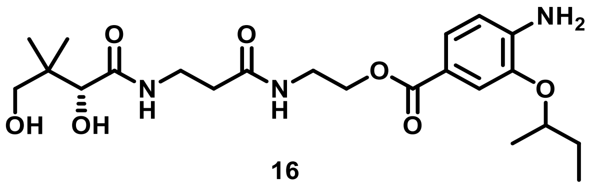

16

Yield: 66% over two steps. White foam. ^{1}H NMR (500 MHz, DMSO) δ 8.20 – 8.04 (m, 1H), 7.79 – 7.64 (m, 1H), 7.39 (d, *J* = 8.2 Hz, 1H), 7.31 (s, 1H), 6.65 (d, *J* = 8.2 Hz, 1H), 5.53 (s, 2H), 5.36 (d, *J* = 5.3 Hz, 1H), 4.46 (t, *J* = 5.2 Hz, 1H), 4.39 – 4.28 (m, 1H), 4.14 (t, *J* = 5.1 Hz, 2H), 3.70 (d, *J* = 5.3 Hz, 1H), 3.50 – 3.20 (m, 7H), 3.20 – 3.14 (m, 1H), 2.29 (t, *J* = 6.7 Hz, 2H), 1.78 – 1.54 (m, 2H), 1.24 (d, *J* = 5.8 Hz, 3H), 0.94 (t, *J* = 7.3 Hz, 3H), 0.80 (s, 3H), 0.77 (s, 3H); ^{13}C NMR (126 MHz, DMSO) δ 173.30, 171.22, 166.38, 144.84, 143.58, 124.51, 116.60, 114.42, 112.82, 75.72, 75.44, 68.49, 63.08, 38.20, 35.60, 35.25, 29.02, 21.37, 20.75, 19.51, 10.06; **HRMS**: calculated for $C_{22}H_{36}N_3O_7^+$ [M+H$^+$]: 454.25478; found: 454.25488.

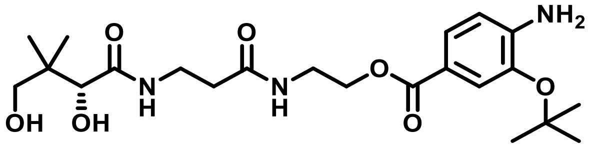

17

Yield: 66% over two steps. White foam. ^{1}H NMR (500 MHz, DMSO) δ 8.23 – 8.03 (m, 1H), 7.70 (t, *J* = 5.1 Hz, 1H), 7.47 (d, *J* = 8.3 Hz, 1H), 7.42 (s, 1H), 6.68 (d, *J* = 8.3 Hz, 1H), 5.61 (s, 2H), 5.36 (d, *J* = 5.3 Hz, 1H), 4.46 (t, *J* = 5.2 Hz, 1H), 4.14 (t, *J* = 5.0 Hz, 2H), 3.70 (d, *J* = 5.2 Hz, 1H), 3.46 – 3.20 (m, 7H), 3.20 – 3.14 (m, 1H), 2.29 (t, *J* = 6.8 Hz, 2H), 1.33 (s, 9H), 0.80 (s, 3H), 0.77 (s, 3H); ^{13}C NMR (126 MHz, DMSO) δ 173.29, 171.21, 166.24, 148.67, 140.57, 126.67, 124.26, 116.24, 113.67, 79.96, 75.44, 68.50, 63.03, 38.22, 35.56, 35.24, 28.80, 21.37, 20.76; **HRMS**: calculated for $C_{22}H_{36}N_3O_7^+$ [M+H$^+$]: 454.25478; found: 454.25490.

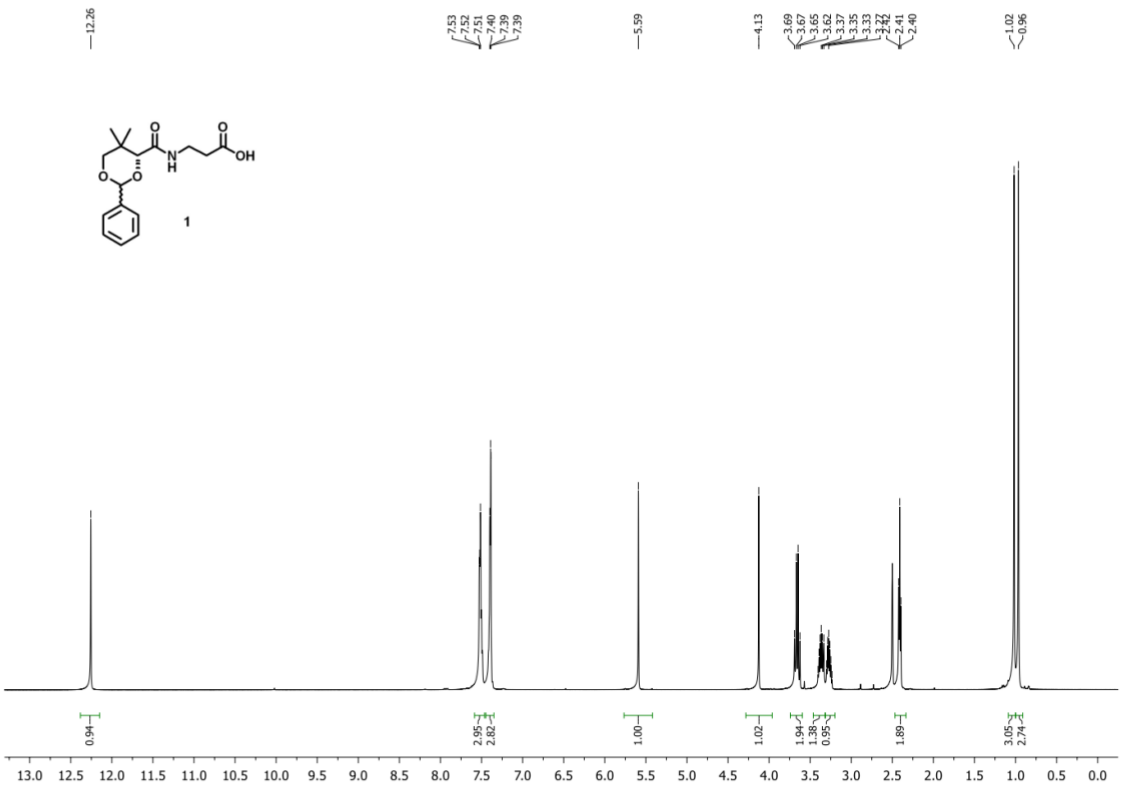
12.26
7.53 7.52 7.51 7.40 7.39 7.39
5.59
4.13
3.69 3.67 3.65 3.62 3.37 3.35 3.33 3.27 2.42 2.41 2.40
1.02 0.96
OH
N
H
O
1
0.94 2.95 2.82 1.00 1.02 1.94 1.38 0.95 1.89 3.05 2.74
13.0 12.5 12.0 11.5 11.0 10.5 10.0 9.5 9.0 8.5 8.0 7.5 7.0 6.5 6.0 5.5 5.0 4.5 4.0 3.5 3.0 2.5 2.0 1.5 1.0 0.5 0.0

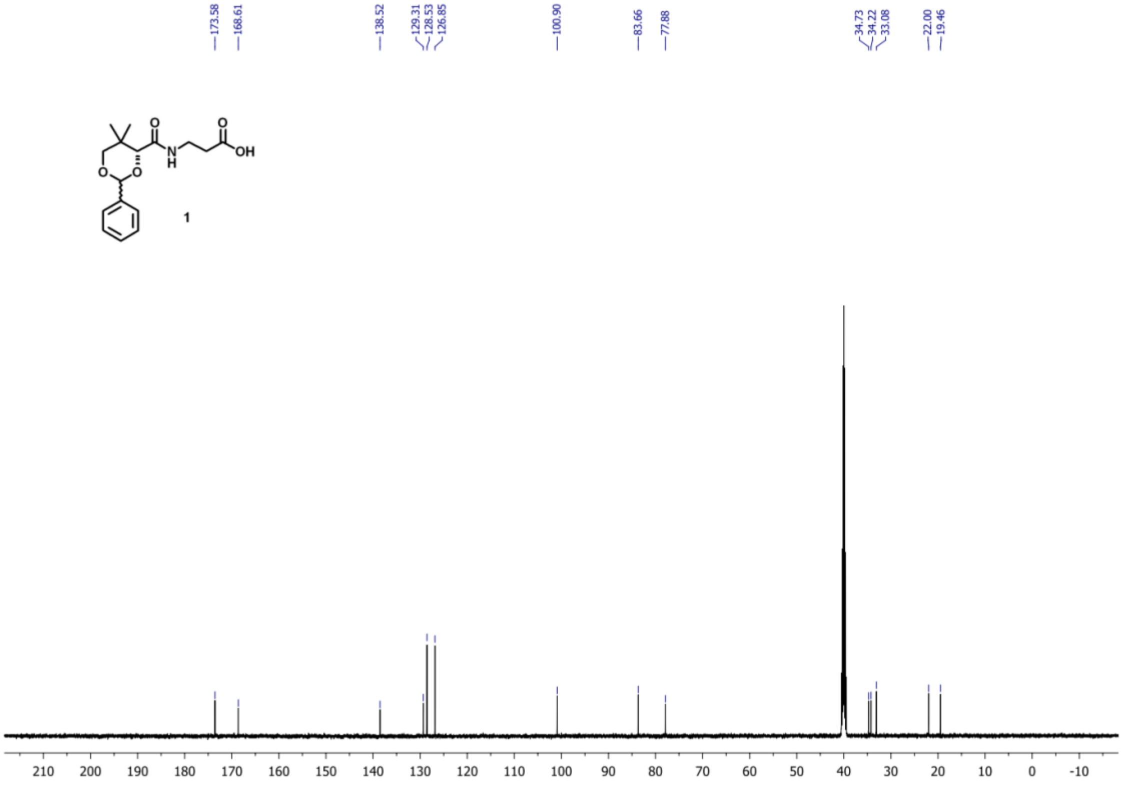
173.58 168.61
138.52
129.31 128.53 126.85
100.90
83.66 77.88
34.73 34.22 33.08
22.00 19.46
OH
N
H
O
1
210 200 190 180 170 160 150 140 130 120 110 100 90 80 70 60 50 40 30 20 10 0 -10

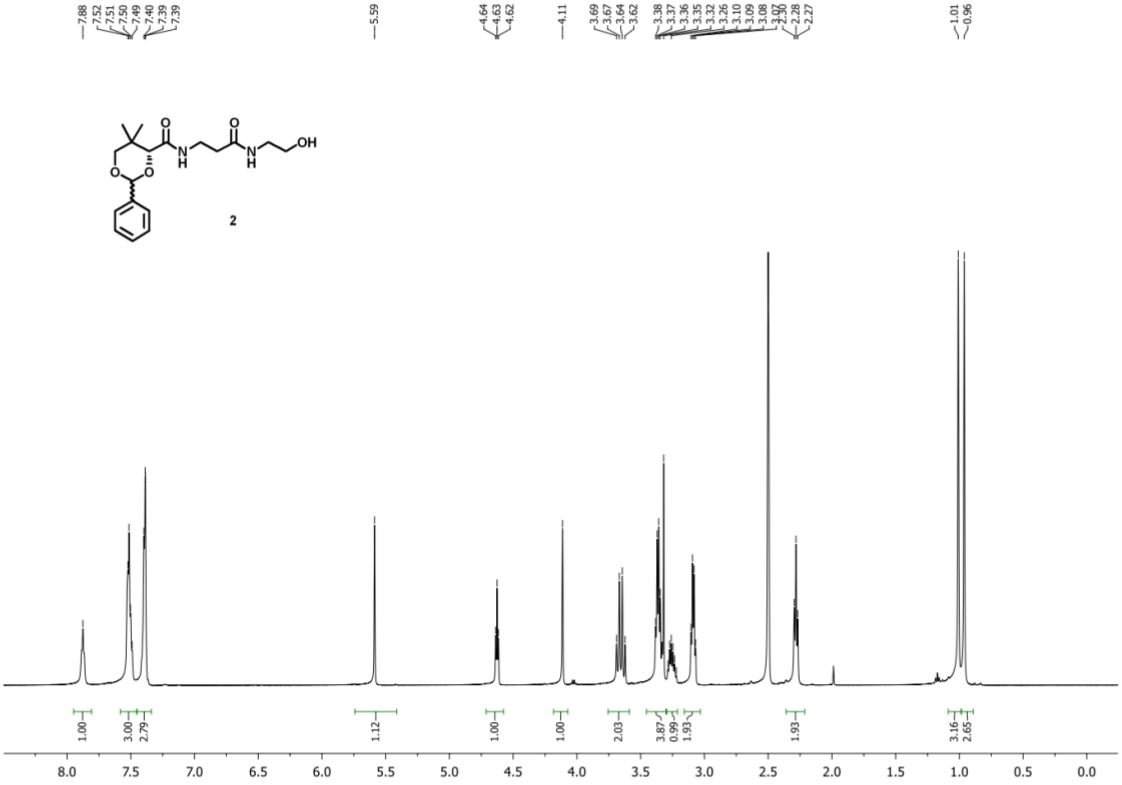
7.88
7.52
7.51
7.50
7.49
7.40
7.39
7.39
5.59
4.64
4.63
4.62
4.11
3.69
3.67
3.64
3.62
3.38
3.37
3.36
3.35
3.32
3.26
3.10
3.09
3.08
2.28
2.27
1.01
0.96
OH
O
N
H
2
1.00
3.00
2.79
1.12
1.00
1.00
2.03
3.87
0.99
1.93
1.93
3.16
2.65
8.0 7.5 7.0 6.5 6.0 5.5 5.0 4.5 4.0 3.5 3.0 2.5 2.0 1.5 1.0 0.5 0.0

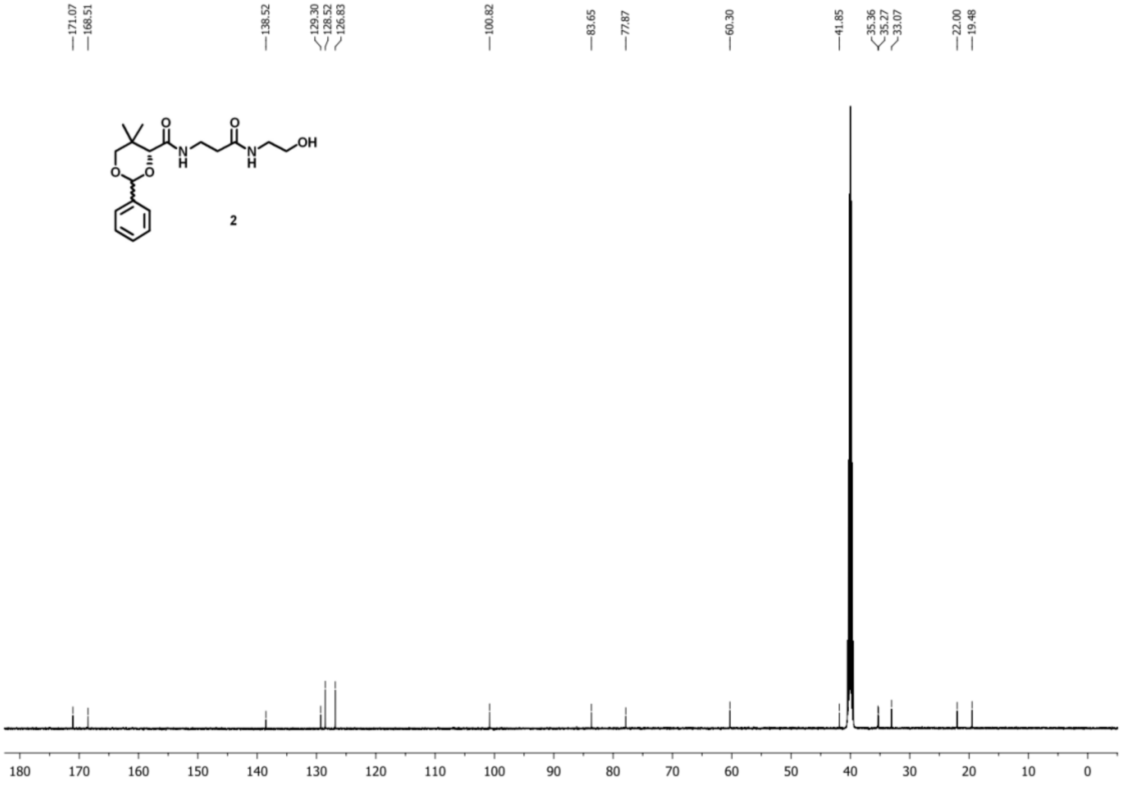
171.07
168.51
138.52
129.30
128.52
126.83
100.82
83.65
77.87
60.30
41.85
35.36
35.27
33.07
22.00
19.48
OH
O
N
H
2
180 170 160 150 140 130 120 110 100 90 80 70 60 50 40 30 20 10 0

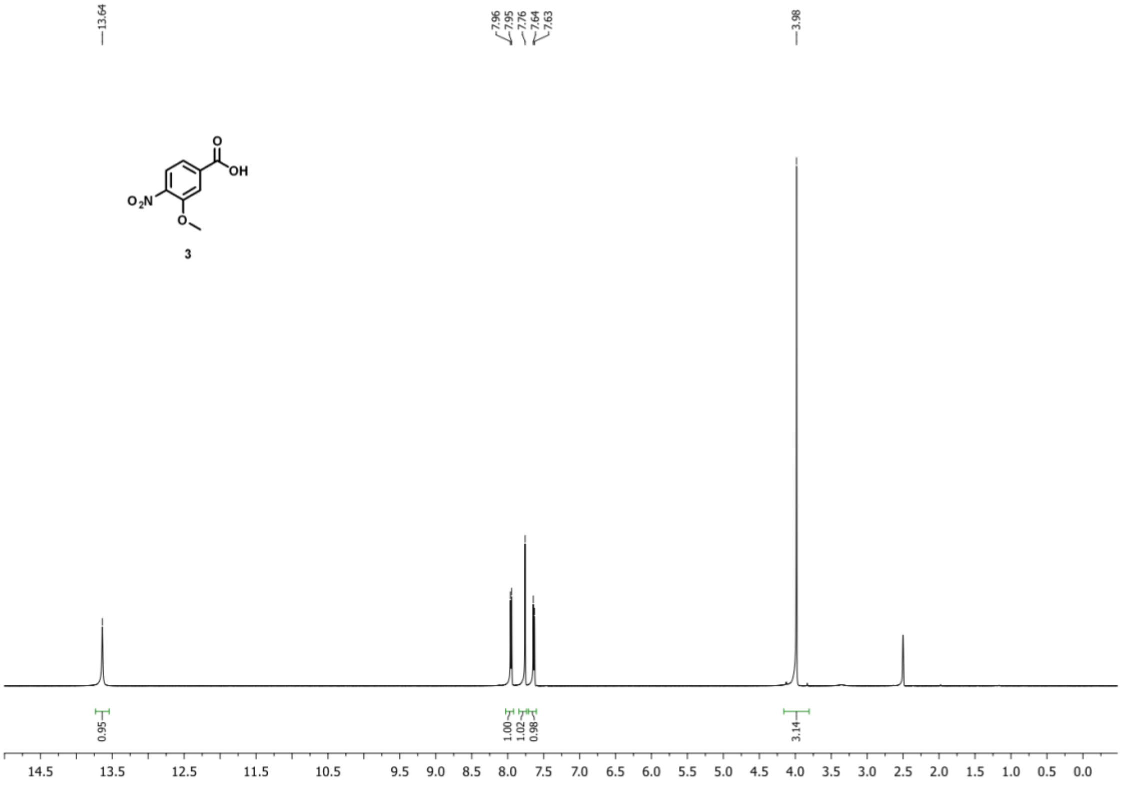
13.64
7.96
7.95
7.76
7.64
7.63
3.98
O
OH
O_2N
O
3
0.95
1.00
1.02
0.98
3.14
14.5 13.5 12.5 11.5 10.5 9.5 9.0 8.5 8.0 7.5 7.0 6.5 6.0 5.5 5.0 4.5 4.0 3.5 3.0 2.5 2.0 1.5 1.0 0.5 0.0

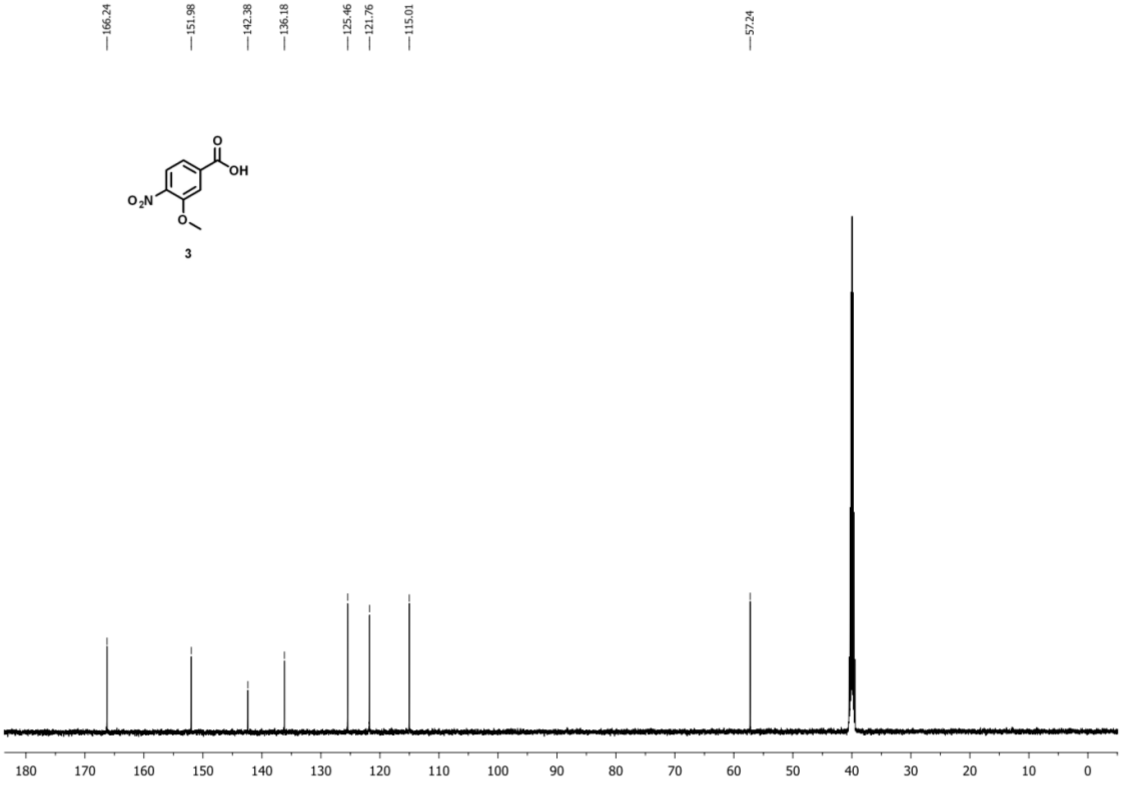
166.24
151.98
142.38
136.18
125.46
121.76
115.01
57.24
O
OH
O_2N
O
3
180 170 160 150 140 130 120 110 100 90 80 70 60 50 40 30 20 10 0

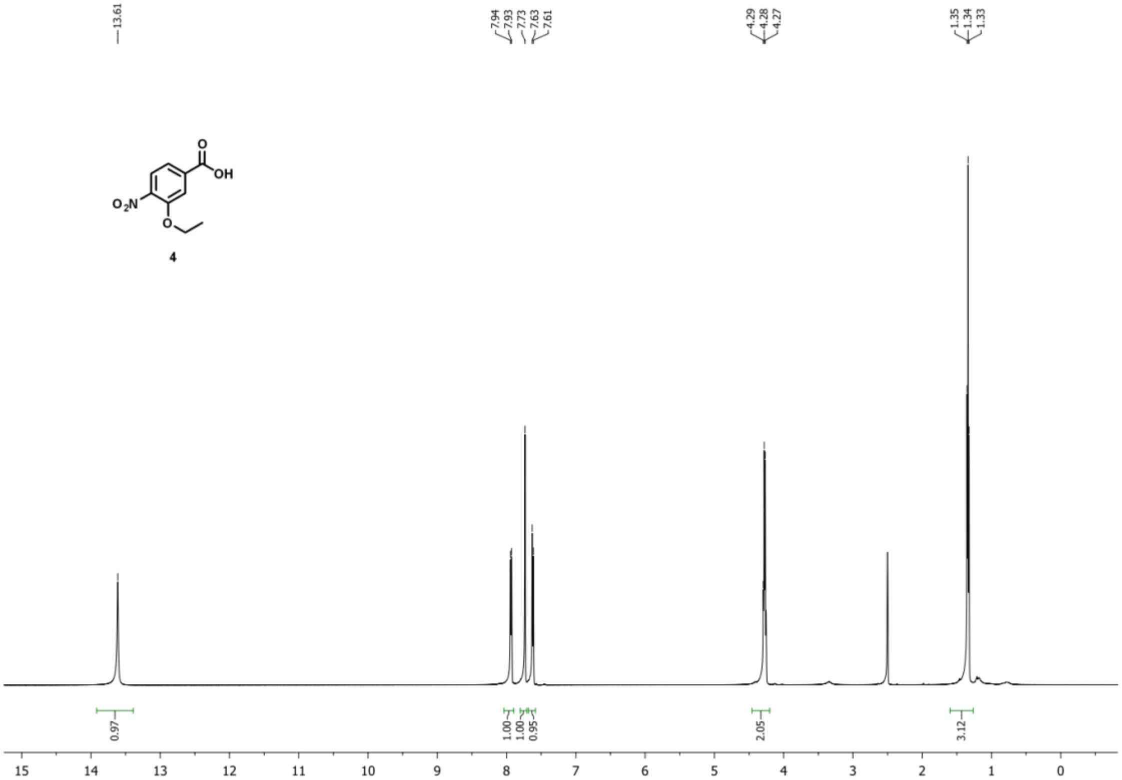
13.61
7.94
7.93
7.73
7.63
7.61
4.29
4.28
4.27
1.35
1.34
1.33
OH
O
O2N
O
4
0.97
1.00
1.00
0.95
2.05
3.12
15 14 13 12 11 10 9 8 7 6 5 4 3 2 1 0

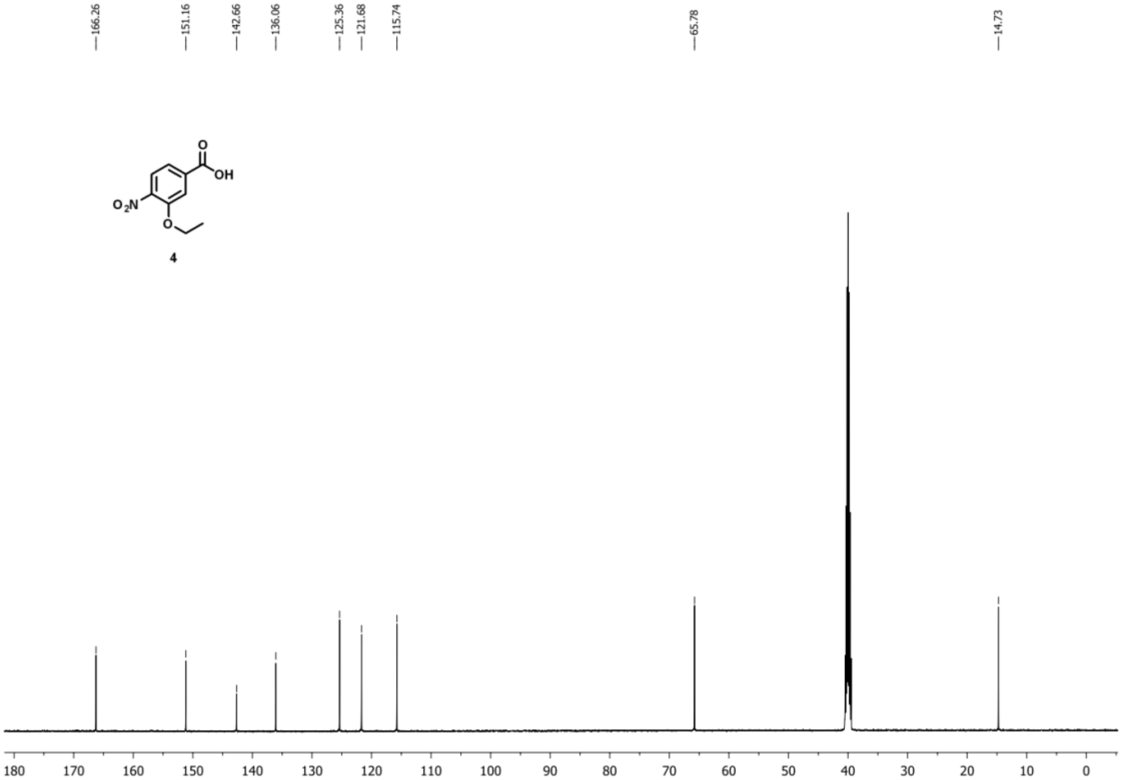
166.26
151.16
142.66
136.06
125.36
121.68
115.74
65.78
14.73
OH
O
O2N
O
4
180 170 160 150 140 130 120 110 100 90 80 70 60 50 40 30 20 10 0

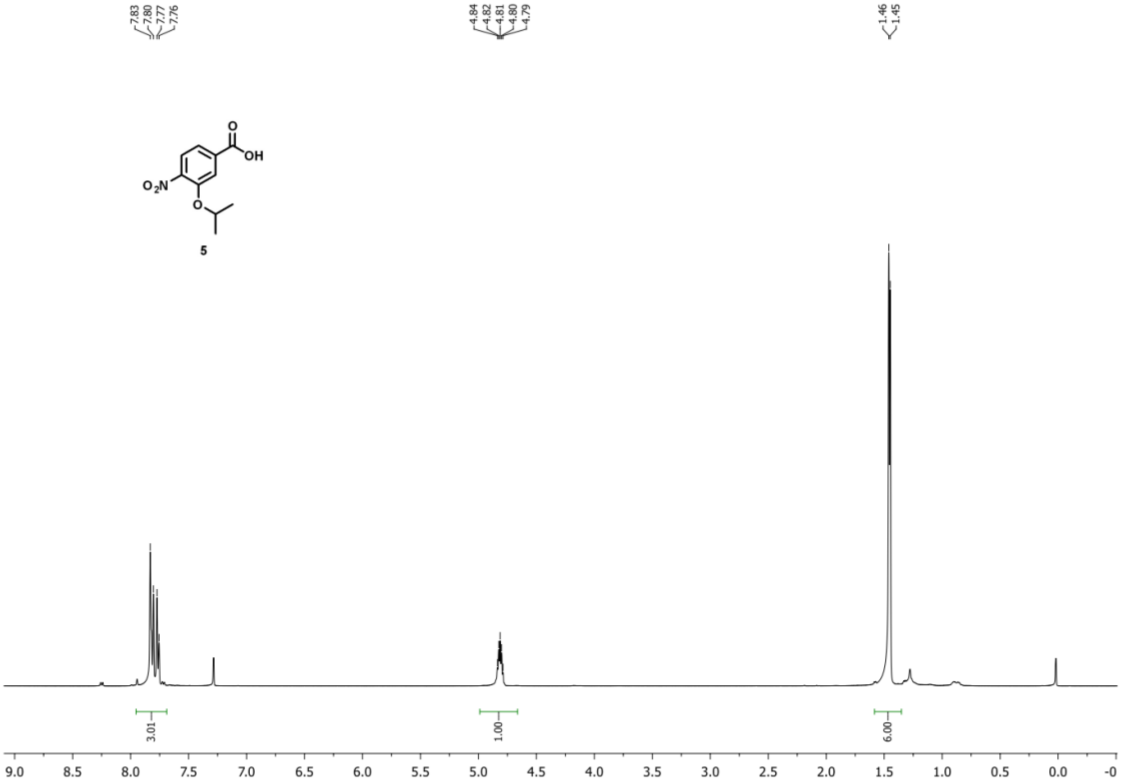
7.83
7.80
7.77
7.76
4.84
4.82
4.81
4.80
4.79
1.46
1.45
O
OH
O_2N
O
5
3.01
1.00
6.00
9.0 8.5 8.0 7.5 7.0 6.5 6.0 5.5 5.0 4.5 4.0 3.5 3.0 2.5 2.0 1.5 1.0 0.5 0.0 -0

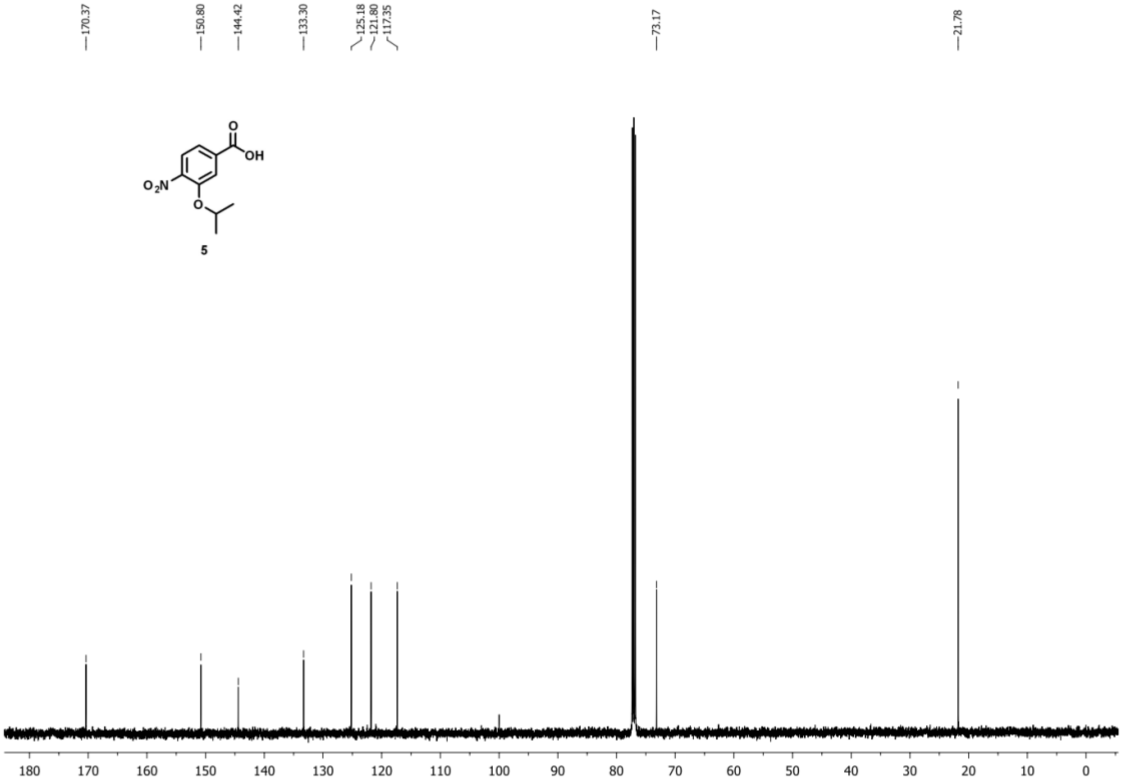
170.37
150.80
144.42
133.30
125.18
121.80
117.35
73.17
21.78
O
OH
O_2N
O
5
180 170 160 150 140 130 120 110 100 90 80 70 60 50 40 30 20 10 0

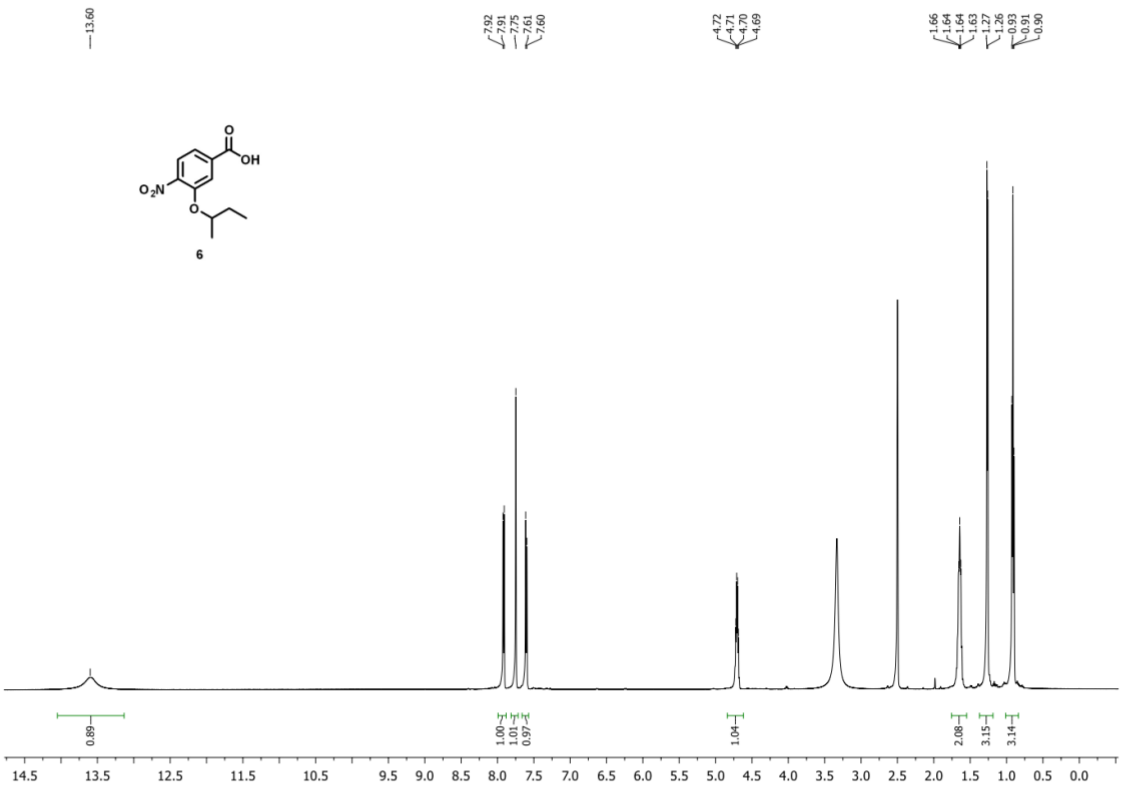

13.60
7.92
7.91
7.75
7.61
7.60
4.72
4.71
4.70
4.69
1.66
1.64
1.64
1.63
1.27
1.26
0.93
0.91
0.90
O
OH
O_2N
O
6
0.89
1.00
1.01
0.97
1.04
2.08
3.15
3.14
14.5 13.5 12.5 11.5 10.5 9.5 9.0 8.5 8.0 7.5 7.0 6.5 6.0 5.5 5.0 4.5 4.0 3.5 3.0 2.5 2.0 1.5 1.0 0.5 0.0

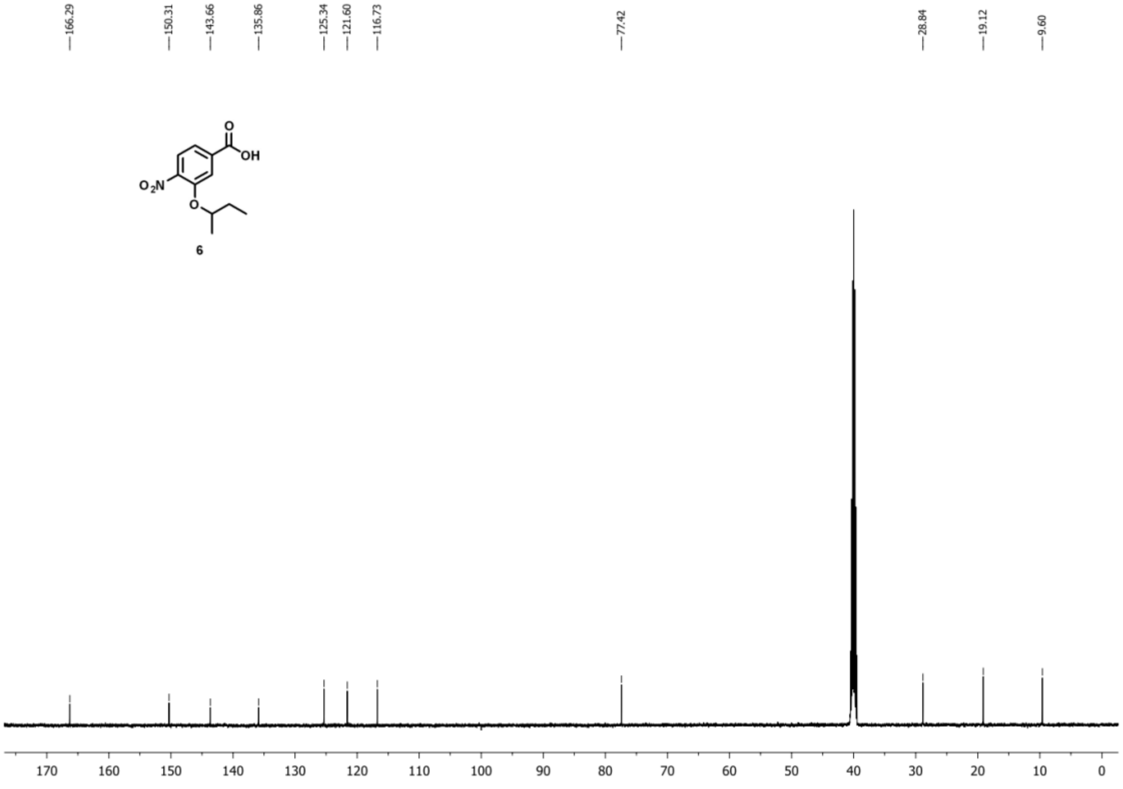

166.29
150.31
143.66
135.86
125.34
121.60
116.73
77.42
28.84
19.12
9.60
O
OH
O_2N
O
6
170 160 150 140 130 120 110 100 90 80 70 60 50 40 30 20 10 0

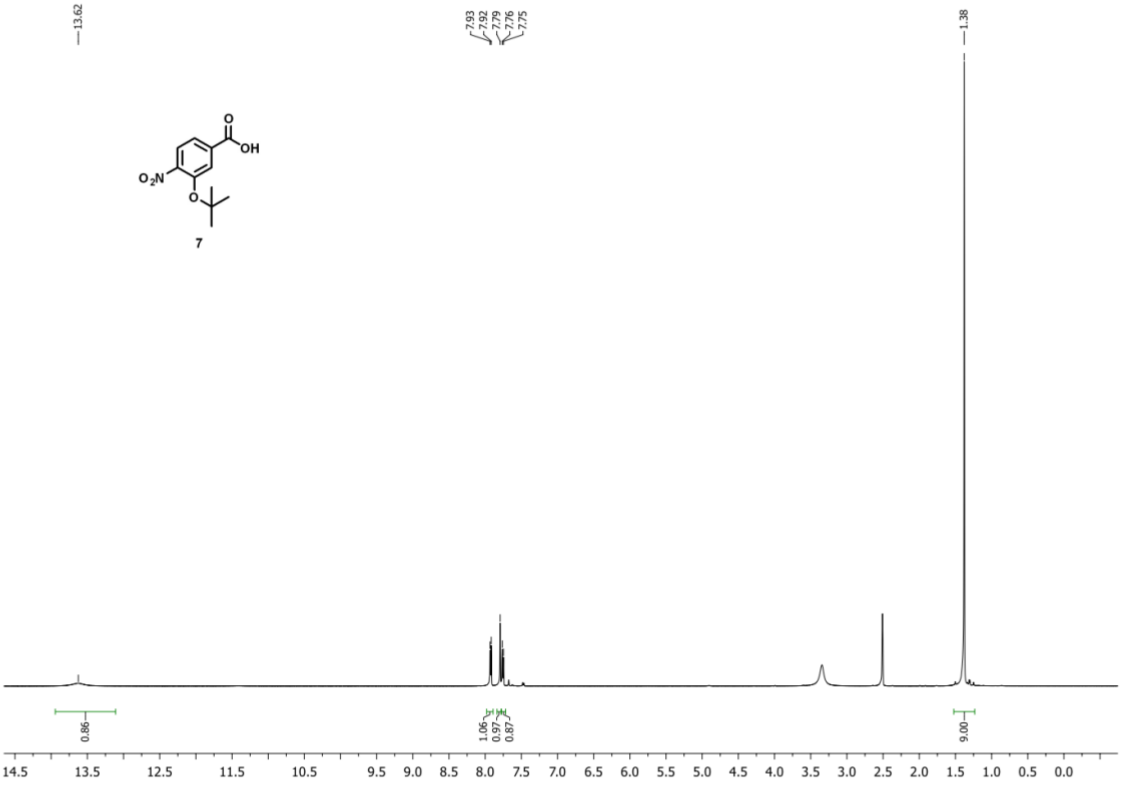
13.62
7.93
7.92
7.79
7.76
7.75
1.38
OH
O
O_2N
7
0.86
1.06
0.97
0.87
9.00
14.5 13.5 12.5 11.5 10.5 9.5 9.0 8.5 8.0 7.5 7.0 6.5 6.0 5.5 5.0 4.5 4.0 3.5 3.0 2.5 2.0 1.5 1.0 0.5 0.0

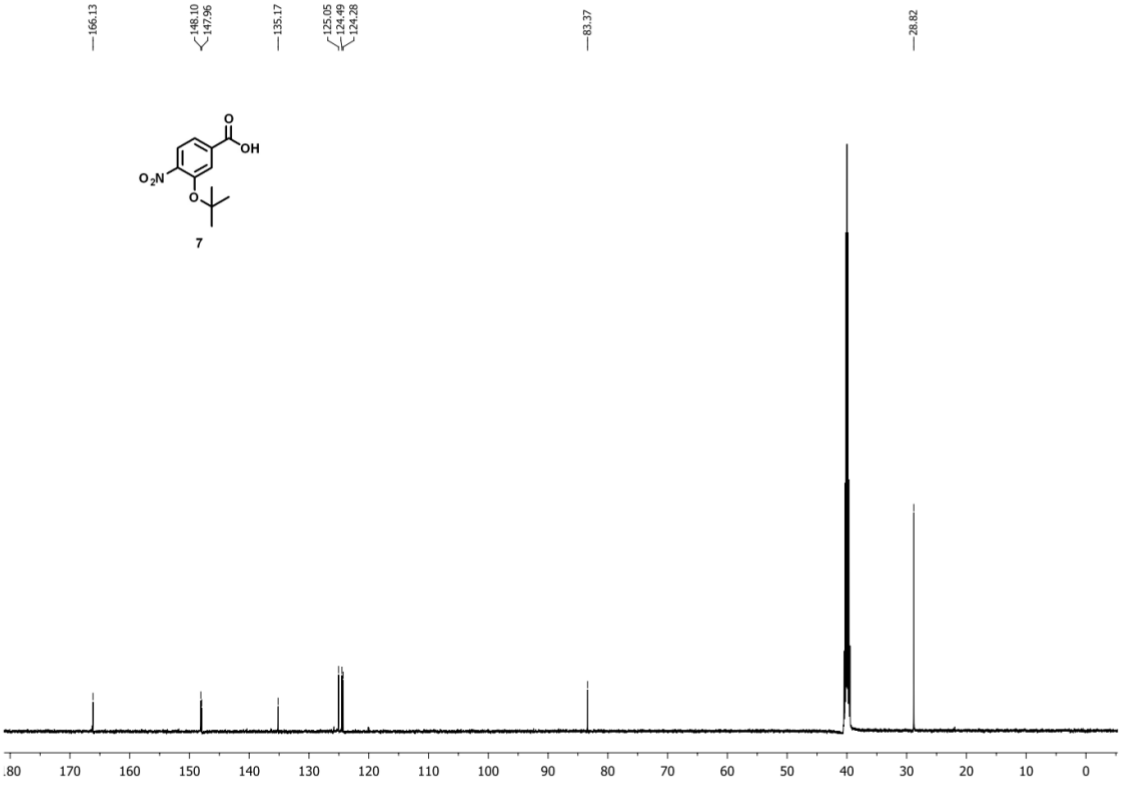
166.13
148.10
147.96
135.17
125.05
124.49
124.28
83.37
28.82
OH
O
O_2N
7
180 170 160 150 140 130 120 110 100 90 80 70 60 50 40 30 20 10 0

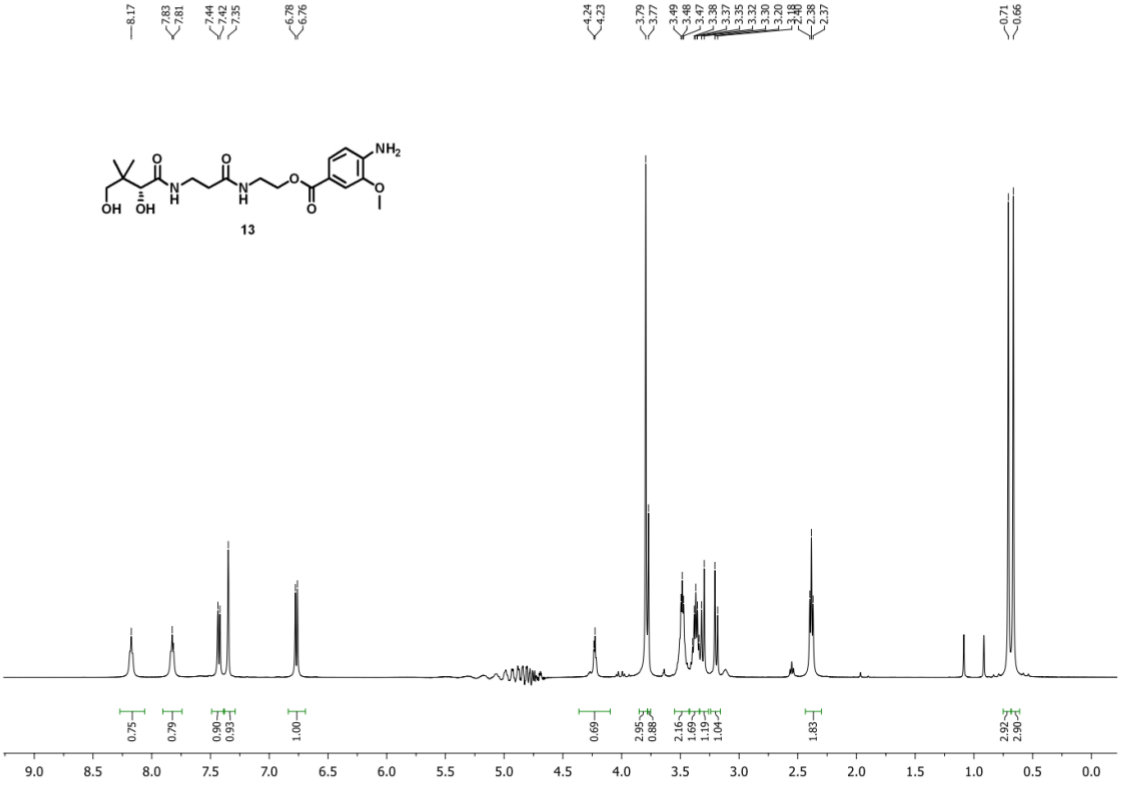
8.17
7.83
7.81
7.44
7.42
7.35
6.78
6.76
4.24
4.23
3.79
3.77
3.49
3.48
3.47
3.38
3.37
3.35
3.32
3.30
3.20
3.18
2.40
2.38
2.37
0.71
0.66
NH2
OH
OH
H
H
13
0.75
0.79
0.90
0.93
1.00
0.69
2.95
0.88
2.16
1.69
1.19
1.04
1.83
2.92
2.90
9.0
8.5
8.0
7.5
7.0
6.5
6.0
5.5
5.0
4.5
4.0
3.5
3.0
2.5
2.0
1.5
1.0
0.5
0.0

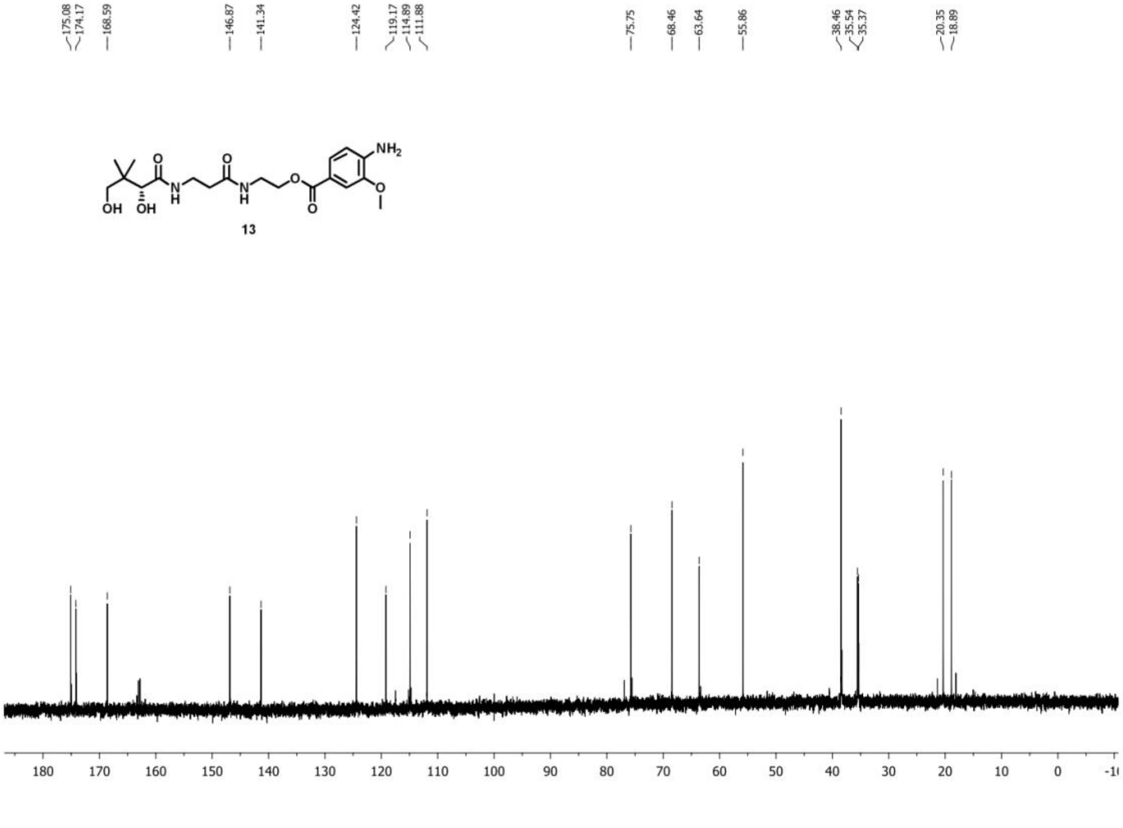
175.08
174.17
168.59
146.87
141.34
124.42
119.17
114.89
111.88
75.75
68.46
63.64
55.86
38.46
35.54
35.37
20.35
18.89
NH2
OH
OH
H
H
13
180
170
160
150
140
130
120
110
100
90
80
70
60
50
40
30
20
10
0

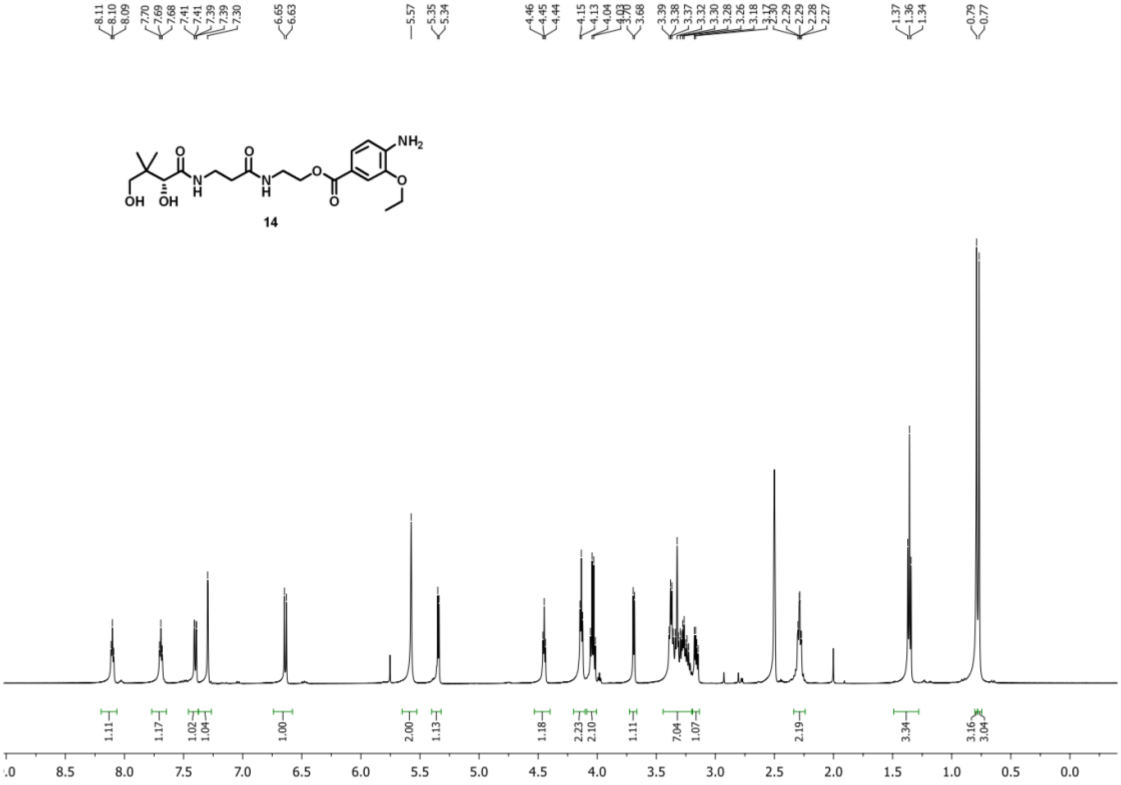

8.11 8.10 8.09 7.70 7.69 7.68 7.41 7.41 7.39 7.39 7.30 6.65 6.63 5.57 5.35 5.34 4.46 4.45 4.44 4.15 4.13 4.04 4.03 3.70 3.68 3.39 3.38 3.37 3.32 3.30 3.28 3.26 3.18 3.17 2.30 2.29 2.29 2.28 2.27 1.37 1.36 1.34 0.79 0.77
NH2
O
O
N H
N H
OH
OH
14
1.11 1.17 1.02 1.04 1.00 2.00 1.13 1.18 2.23 2.10 1.11 7.04 1.07 2.19 3.34 3.16 3.04
.0 8.5 8.0 7.5 7.0 6.5 6.0 5.5 5.0 4.5 4.0 3.5 3.0 2.5 2.0 1.5 1.0 0.5 0.0

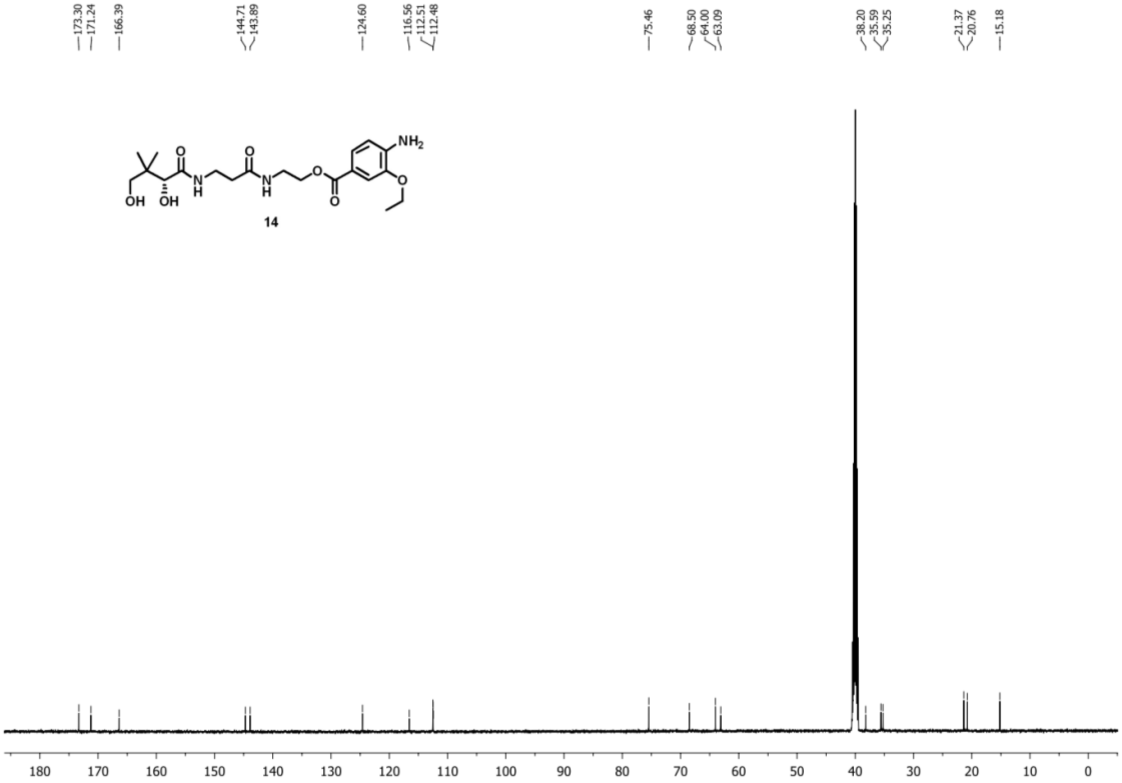

173.30 171.24 166.39 144.71 143.89 124.60 116.56 112.51 112.48 75.46 68.50 64.00 63.09 38.20 35.59 35.25 21.37 20.76 15.18
NH2
O
O
N H
N H
OH
OH
14
180 170 160 150 140 130 120 110 100 90 80 70 60 50 40 30 20 10 0

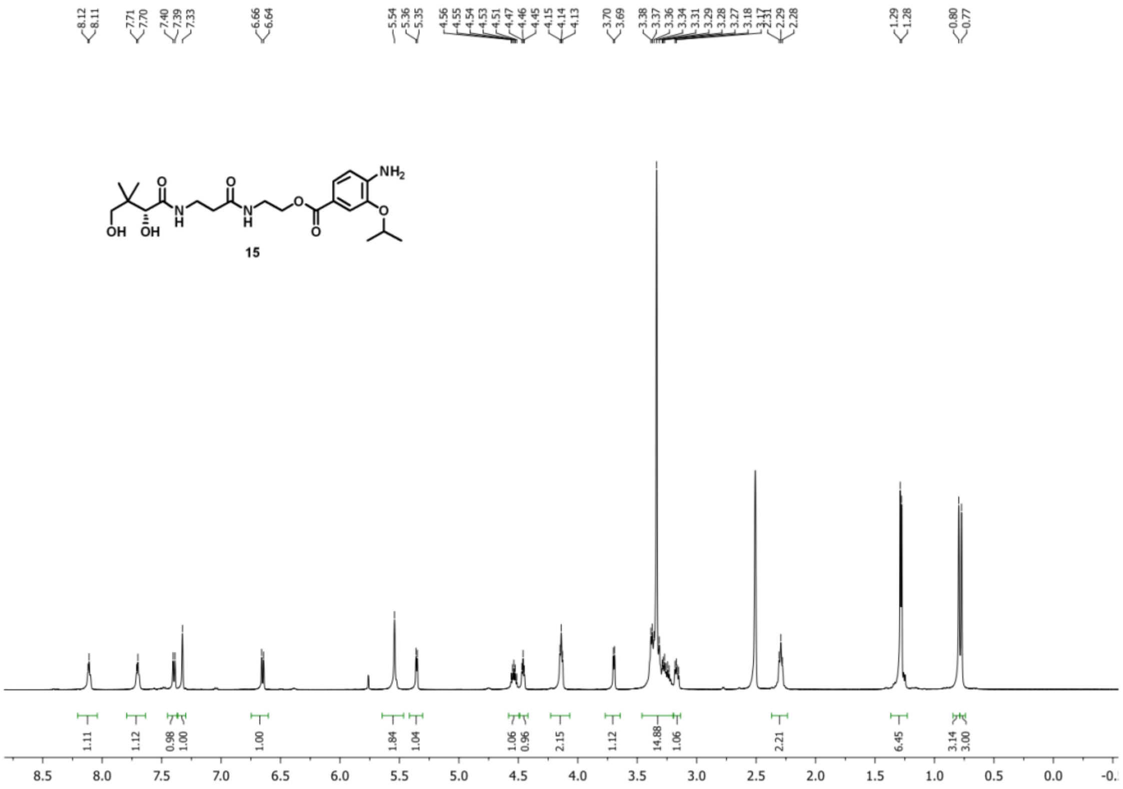
8.12
8.11
7.71
7.70
7.40
7.39
7.33
6.66
6.64
5.54
5.36
5.35
4.56
4.55
4.54
4.53
4.51
4.47
4.46
4.45
4.15
4.14
4.13
3.70
3.69
3.38
3.37
3.36
3.34
3.31
3.29
3.28
3.27
3.18
3.17
2.31
2.29
2.28
1.29
1.28
0.80
0.77
NH2
O
N
H
OH
OH
15
1.11
1.12
0.98
1.00
1.00
1.84
1.04
1.06
0.96
2.15
1.12
14.88
1.06
2.21
6.45
3.14
3.00
8.5
8.0
7.5
7.0
6.5
6.0
5.5
5.0
4.5
4.0
3.5
3.0
2.5
2.0
1.5
1.0
0.5
0.0

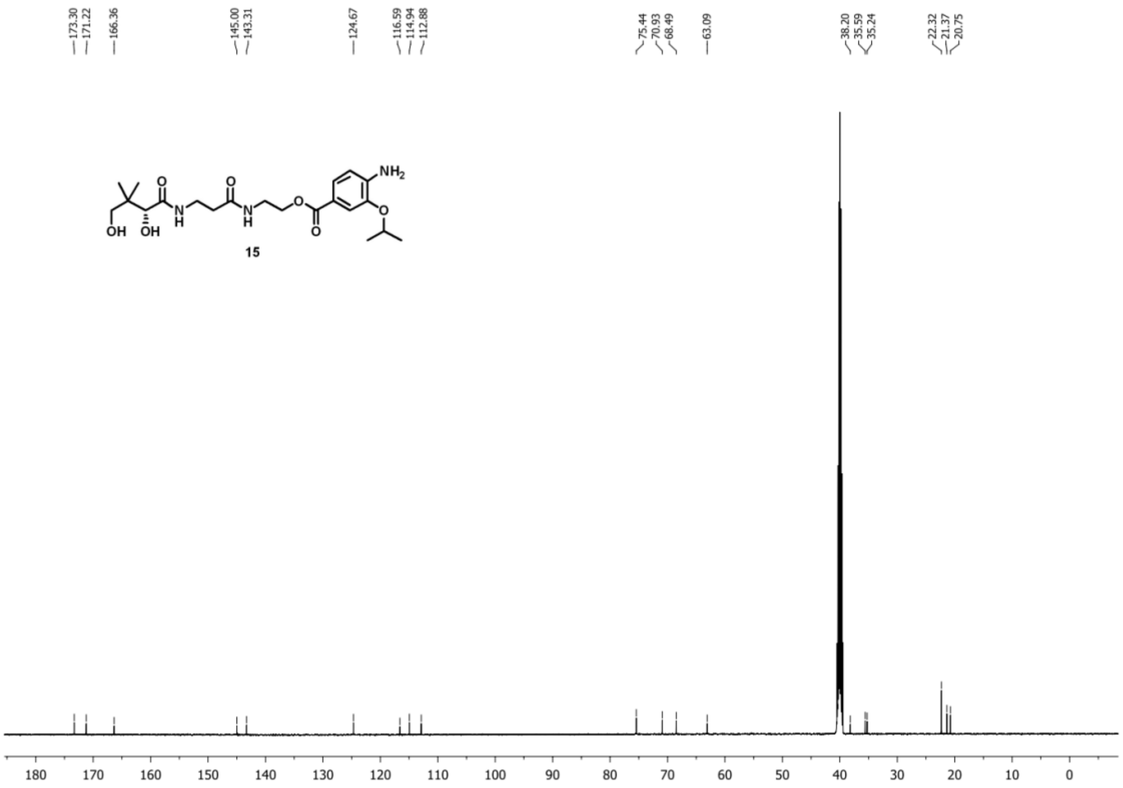
173.30
171.22
166.36
145.00
143.31
124.67
116.59
114.94
112.88
75.44
70.93
68.49
63.09
38.20
35.59
35.24
22.32
21.37
20.75
NH2
O
N
H
OH
OH
15
180
170
160
150
140
130
120
110
100
90
80
70
60
50
40
30
20
10
0

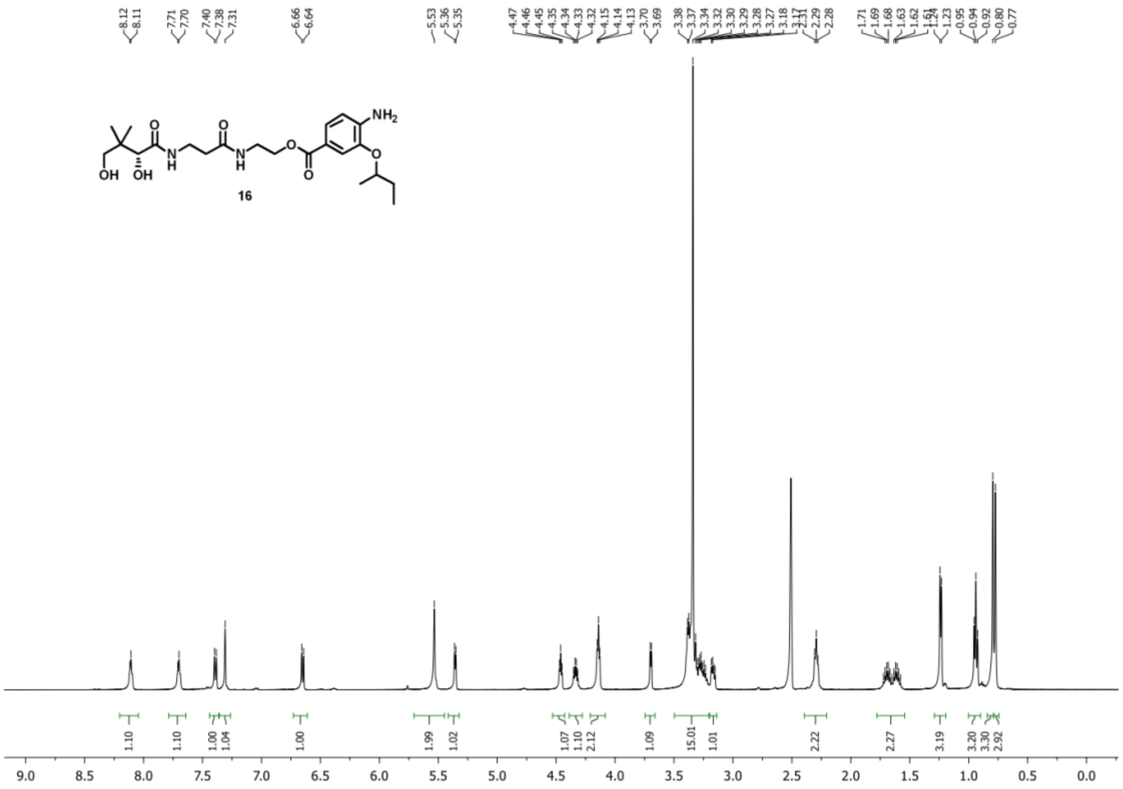
NH2
OH
OH
H
16
9.0 8.5 8.0 7.5 7.0 6.5 6.0 5.5 5.0 4.5 4.0 3.5 3.0 2.5 2.0 1.5 1.0 0.5 0.0

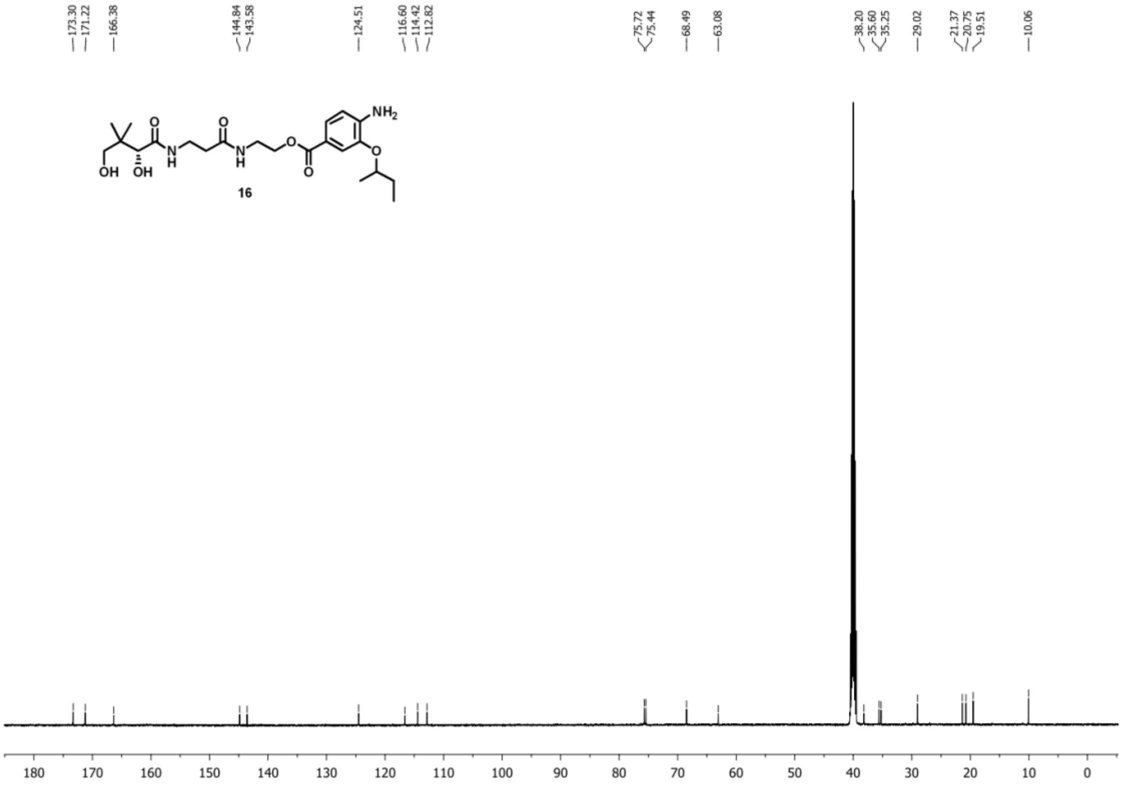
NH2
OH
OH
H
16
180 170 160 150 140 130 120 110 100 90 80 70 60 50 40 30 20 10 0

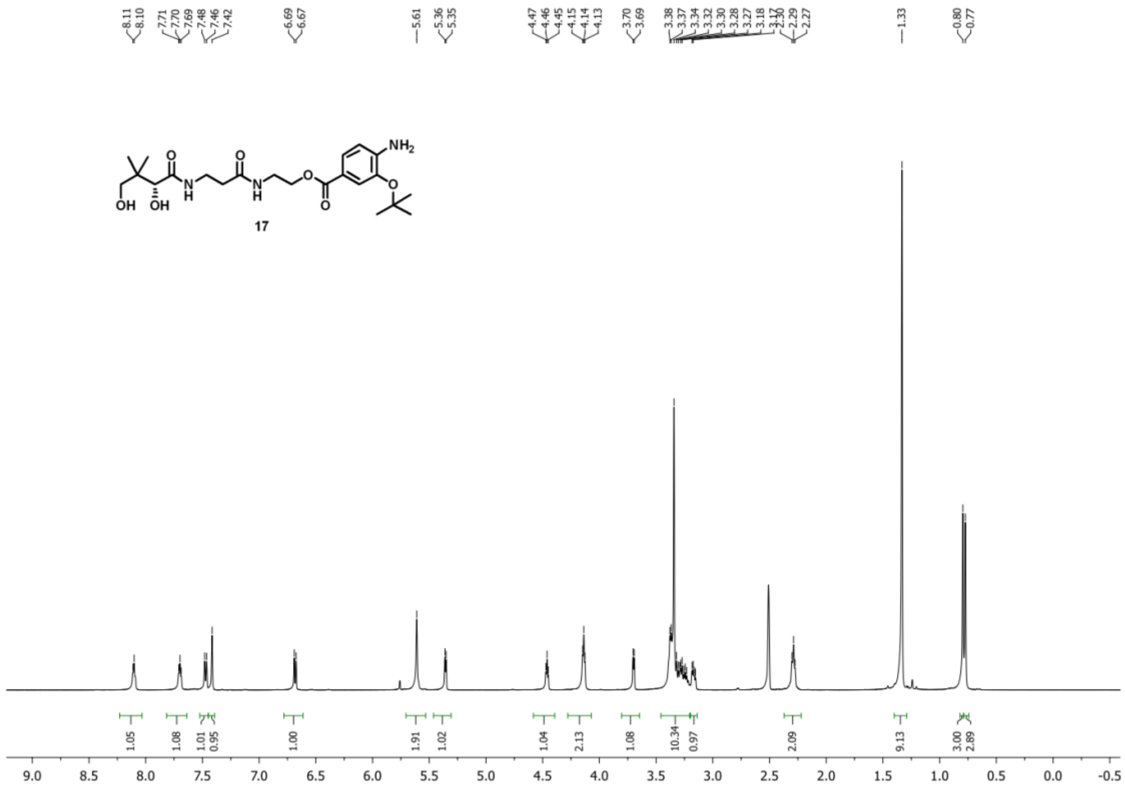
8.11
8.10
7.71
7.70
7.69
7.48
7.46
7.42
6.69
6.67
5.61
5.36
5.35
4.47
4.46
4.45
4.15
4.14
4.13
3.70
3.69
3.38
3.37
3.34
3.32
3.30
3.28
3.27
3.18
3.17
2.30
2.29
2.27
1.33
0.80
0.77
NH2
OH
OH
H
N
O
17
1.05
1.08
1.01
0.95
1.00
1.91
1.02
1.04
2.13
1.08
10.34
0.97
2.09
9.13
3.00
2.89
9.0
8.5
8.0
7.5
7.0
6.5
6.0
5.5
5.0
4.5
4.0
3.5
3.0
2.5
2.0
1.5
1.0
0.5
0.0
-0.5

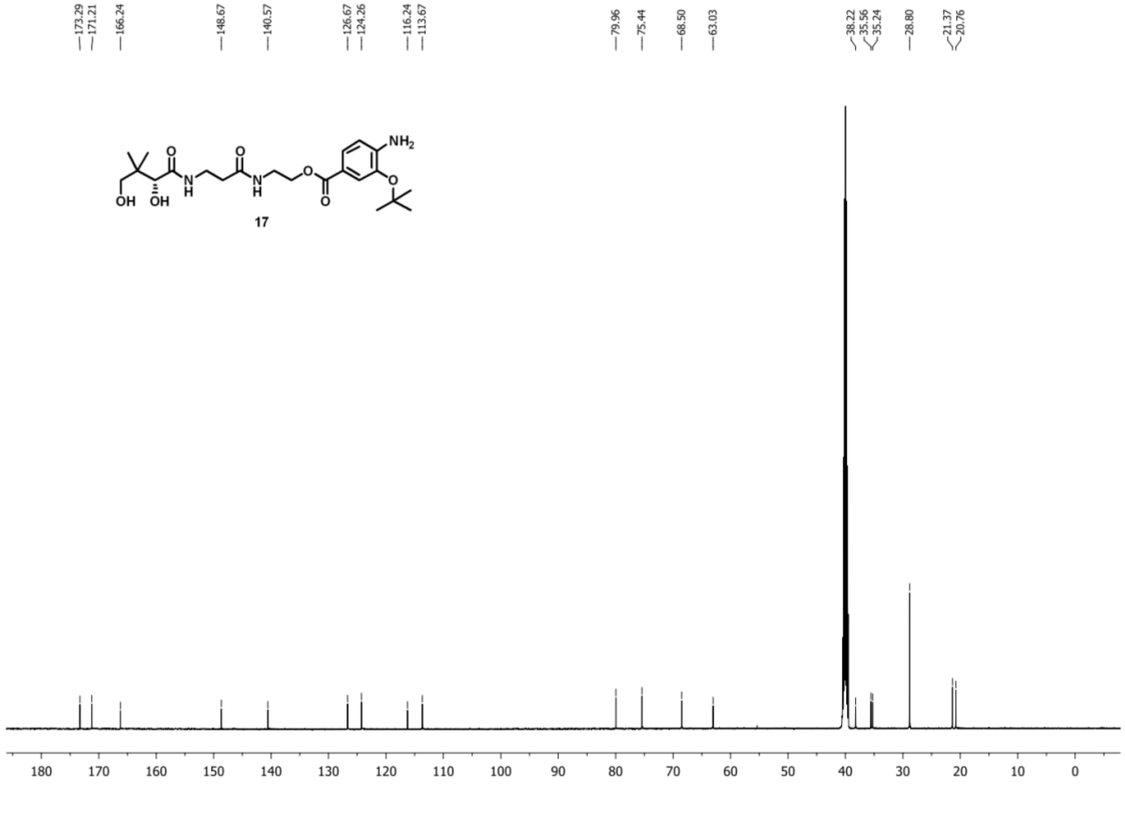
173.29
171.21
166.24
148.67
140.57
126.67
124.26
116.24
113.67
79.96
75.44
68.50
63.03
38.22
35.56
35.24
28.80
21.37
20.76
NH2
OH
OH
H
N
O
17
180
170
160
150
140
130
120
110
100
90
80
70
60
50
40
30
20
10
0
